# Breaking the Air–Water Paradigm: Ion Behavior at Hydrophobic Solid–Water Interfaces

**DOI:** 10.1021/jacs.5c20209

**Published:** 2026-03-19

**Authors:** Xavier R. Advincula, Kara D. Fong, Yongkang Wang, Christoph Schran, Mischa Bonn, Angelos Michaelides, Yair Litman

**Affiliations:** † Yusuf Hamied Department of Chemistry, 2152 University of Cambridge, Lensfield Road, Cambridge CB2 1EW, U.K.; ‡ Cavendish Laboratory, Department of Physics, University of Cambridge, Cambridge CB3 0HE, U.K.; § Lennard-Jones Centre, University of Cambridge, Trinity Ln, Cambridge CB2 1TN, U.K.; ∥ Division of Chemistry and Chemical Engineering, 6469 California Institute of Technology, Pasadena, California 91125, United States; ⊥ Marcus Center for Theoretical Chemistry, California Institute of Technology, Pasadena, California 91125, United States; # 28308 Max Planck Institute for Polymer Research, Ackermannweg 10, Mainz 55128, Germany

## Abstract

Hydrophobic solid–water interfaces underpin processes in nanofluidics, electrochemistry, and energy technologies. Microscopic insights into these systems are often inferred from our understanding of the air–water interface, which is assumed to exhibit similar behavior. Here, we challenge this paradigm by combining heterodyne-detected vibrational sum-frequency generation spectroscopy with machine-learning molecular dynamics simulations at first-principles accuracy to investigate the graphene–NaCl­(aq) interface as a prototypical hydrophobic solid–water system. Spectroscopic results suggest that ions have a minimal effect on the structure of the interfacial water, while simulations reveal that Na^+^ and Cl^–^ accumulate densely at the surface. Together, these findings reveal a new adsorption mechanism that departs from the established air–water interface paradigm, where interfacial ion adsorption is typically associated with, and often detected through, a pronounced alteration of the interfacial water alignment and orientation. This difference arises because ions cannot penetrate the solid boundary and reside at a similar depth as the interfacial water molecules. As a consequence, large ion populations can be accommodated within the extended two-dimensional hydrogen-bond network at the interface, causing only minor local distortions but significant changes to its longer-range connectivity. These results reveal a distinct mechanism of electrolyte organization at aqueous–carbon interfaces, relevant to energy applications, where performance is highly sensitive to the local organization of interfacial water.

## Graphical abstract

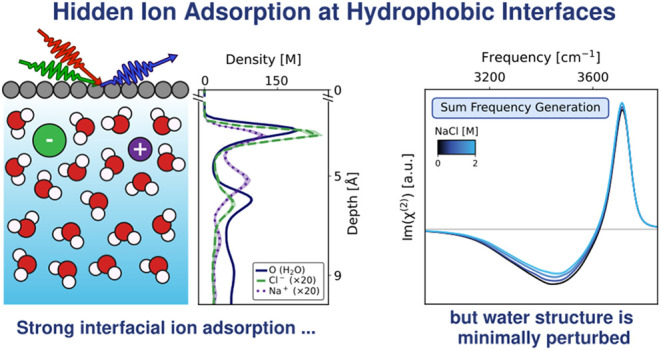

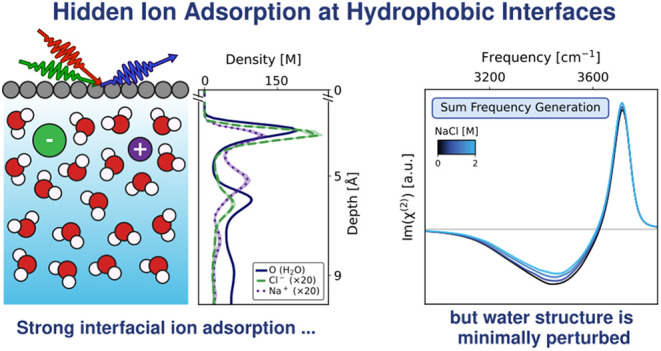

## Introduction

Understanding how ions interact in water near hydrophobic interfaces has puzzled scientists for decades^1–3^ and it lies at the heart of technologies ranging from water purification and desalination^4,5^ and atmospheric chemistry^6–8^ to biomimetic design^9^ and electrochemical energy storage.^10,11^

In this context, a significant body of literature has asserted that the water–air interface is the most common and simple aqueous interface and serves as a reference system to understand water at hydrophobic surfaces,^13–17^ and more specifically, that ion distributions at hydrophobic solid–water interfaces closely mirror those at the air–water boundary. For example, Koelsch et al.^18^ reasoned that key properties of hydrophobic aqueous interfaces, including ion exclusion effects and hydration structure, are fundamentally similar to those observed at the air–water interface, attributing this to a generic hydrophobic effect that governs both environments. Cui et al.^19^ extended this analogy by demonstrating, through simulations, that the potential of mean force (PMF) profiles for chloride and iodide ions at both (hydrophobic) protein and air interfaces were nearly indistinguishable, thereby suggesting that molecular-level organization of ions and water is essentially shared between the two types of surfaces. Furthermore, seminal works by the Saykally and Geissler groups^20,21^ used nonlinear spectroscopic techniques and molecular simulations and determined that SCN^–^ presents a very similar Gibbs adsorption free energy at the air–water, graphene–water, and toluene-water interfaces, even though different mechanisms might be at play. More recently, Scalfi et al.^22^ reported that force-field and *ab initio* simulations yield closely aligned PMF profiles for ions at both air and graphene interfaces, reinforcing the widespread notion that these interfaces are functionally analogous for ion adsorption phenomena. Collectively, these studies suggest that the organization of interfacial water at the water–hydrophobic interface is characterized by relatively weak water–hydrophobe interactions and is dominated by strong water–water interactions. These interactions maximize hydrogen bonding and promote the formation of a two-dimensional network, which underlies the anomalously high surface tension observed for water.^23,24^ In this canonical paradigm, the air–water interface can thus be regarded as the limiting case, where water–hydrophobe interactions vanish. Accordingly, the presence of ions at hydrophobic interfaces is expected to disrupt the interfacial hydrogen-bond network as they do for the air–water interface.^12,25–27^

Hydrophobic interfaces can differ markedly in their mechanical properties. For example, liquid hydrophobic interfaces, such as oil–water boundaries, are characterized by pronounced capillary-wave (CW) fluctuations and partial molecular interpenetration, leading to diffuse interfacial regions and weakened water layering.^21,28,29^ In fact, the air–water interface represents an extreme realization of this fluctuation-dominated regime, where large CW fluctuations enable strong coupling between ions and interfacial fluctuations, influencing properties such as surface tension.^30–32^ By contrast, atomically flat solids such as graphene strongly suppress CW fluctuations and impose long-range geometric constraints.^20,33,34^ Focusing on such rigid solid–water interfaces, therefore, provides a clean route to disentangling hydrophobic effects from interfacial softness and from the molecular identity of the hydrophobic phase Whether paradigms developed primarily from studies of the air–water interface remain valid under these fluctuation-suppressed conditions is a fundamental and open question.

In this work, we show that the canonical view of ion behavior at air–water interfaces does not necessarily apply to solid–water boundaries. Ions can accumulate robustly at hydrophobic carbons without substantially perturbing the local structure of interfacial water. To demonstrate this, we combine heterodyne-detected vibrational sum-frequency generation (HD-VSFG) with machine-learning molecular dynamics simulations. While simple electrolytes such as NaCl exhibit pronounced stratification and ion-depleted surface layers at air–water interfaces,^12,35,36^ our results reveal that the water–graphene interface can host densely adsorbed ions while leaving the hydrogen-bond network largely intact. This marked contrast highlights that ion adsorption onto extended flat hydrophobic solids is primarily driven by subtle hydration structuring rather than by the direct disruption of interfacial water observed at air–water boundaries. Thus, paradigms developed for the air–water interface cannot be simply transferred to solid–water interfaces.

## Results

### Experimental VSFG Spectrum of NaCl­(aq)/Graphene is Indistinguishable from That of NaCl­(aq)/Air

To experimentally probe interfacial water structure in electrolyte solutions, VSFG spectroscopy has proven invaluable, as it is intrinsically surface-specific and highly sensitive to the orientation of interfacial water molecules.^16,37–43^ Although atomic ions lack vibrational modes of their own, their presence can be inferred from the response of surrounding water molecules. Phase-resolved VSFG, realized experimentally through HD-VSFG, extends this capability by providing direct access to the imaginary part of the nonlinear susceptibility, Im­(χ^(2)^), thereby unambiguously isolating the resonant vibrational response of interfacial water from the nonresonant responses.^44–47^ Moreover, the sign of the Im­(χ^(2)^) spectrum reflects the net orientation of water molecules at the interface. For water molecules with effectively uncoupled O–H stretches, such as those possessing a free O–H group, a positive signal corresponds to O–H bonds pointing toward the interface (away from the bulk liquid), whereas a negative signal indicates O–H bonds oriented toward the bulk region.^48,49^ By contrast, for intramolecularly coupled O–H bonds, the signal reports on orientations parallel and perpendicular to the water bisector for the symmetric and asymmetric stretching modes, respectively. In addition, intermolecular vibrational coupling between O–H groups results in a broadening of the hydrogen-bonded O–H band.^50,51^

We first consider the well-established case of HCl solutions, where surface-active protons adsorb strongly at the air–water interface,^25–27^ disrupting the interfacial hydrogen-bond network. As a result, the Im­(χ^(2)^) spectra change dramatically compared to pure water, with pronounced reshaping of the 3,000–3,600 cm^–1^ region, arising from hydrogen–bonded O–H stretches, and suppression of the free O–H peak, corresponding to non-hydrogen-bonded O–H stretches. (Figure 1a).^25,26^ In contrast, when ions are excluded from the surface, as in NaCl solutions, the spectra remain largely similar to that of pure water (Figure 1b).^12^

**Figure 1:**
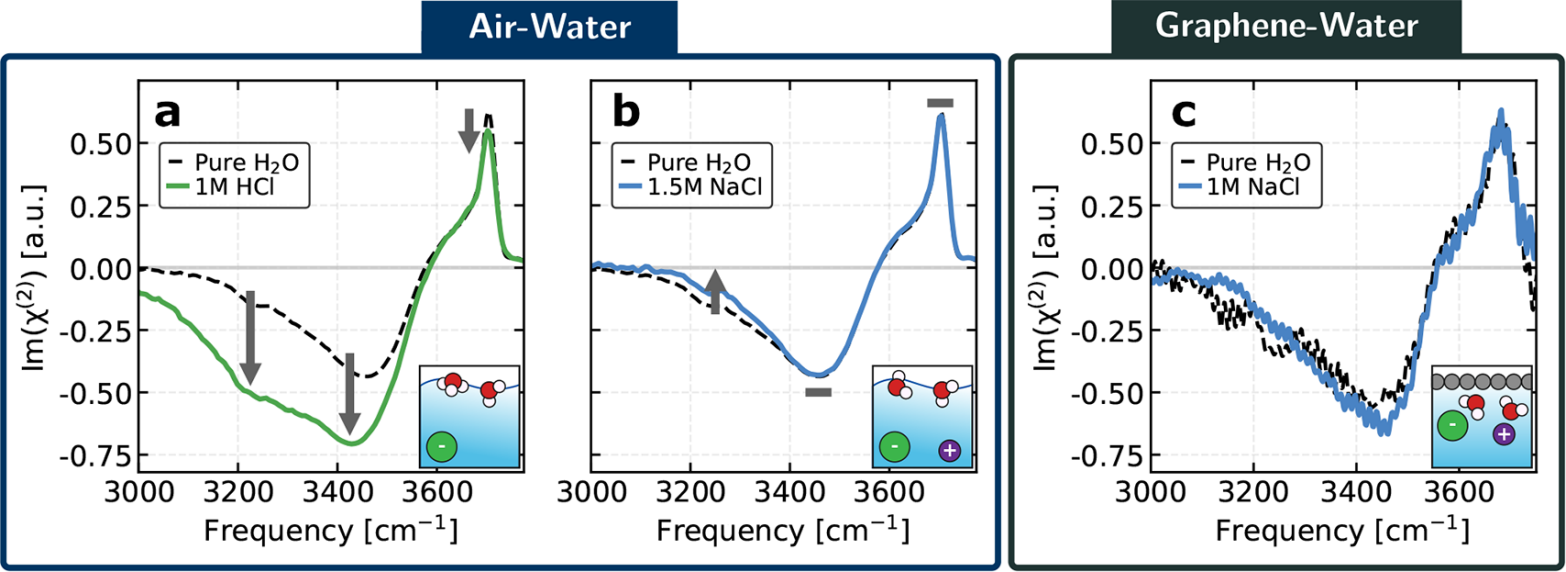
Experimental HD-VSFG spectra of electrolyte solutions at air–liquid and solid–liquid interfaces. HD-VSFG spectra at room temperature for (a) 1.0 M HCl at the air–water interface, (b) 1.5 M NaCl at the air–water interface, and (c) 1.0 M NaCl at the graphene–water interface. In each panel, the corresponding pure-water spectrum for the same interface is shown as a reference. (a) and (b) are adapted with permission from Litman et al.^12^ The arrows indicate the direction of spectral changes with respect to the pure water spectrum, while gray rectangles mark regions where changes are negligible. The accompanying illustrations schematically indicate the presence or absence of ions at each interface.

In Figure 1c, we present the experimental HD-VSFG Im­(χ^(2)^) spectra for a prototypical hydrophobic solid–water interface, the focus of this work, the graphene-NaCl­(aq) interface. The spectrum displays a broad negative band centered between 3,200–3,550 cm^–1^, corresponding to hydrogen-bonded O–H stretches, and a narrow positive peak above 3,600 cm^–1^ associated with the dangling O–H of interfacial water, consistent with previous reports,^47,52^ characteristic of a hydrophobic interface.

Compared to air–water measurements (Figure 1a,b), HD-VSFG at graphene–liquid interfaces is experimentally challenging and generally yields a lower signal-to-noise ratio, as seen in the noisier traces of Figure 1c. Within the experimental uncertainty, both the hydrogen-bonded band and the dangling O–H peak exhibit indistinguishable changes upon NaCl addition at the graphene–water interface. Such weak perturbations might appear to indicate only minor ionic effects, similar to the subtle spectral changes observed at the air–water interface.^53–56^ However, some of us have shown that ions can in fact accumulate at graphitic solid–liquid interfaces.^57^ Reconciling this apparently paradoxical behavior requires going beyond what the experimental spectra alone can reveal.

### Simulations Reproduce the Experimental VSFG Spectrum and Reveal Interfacial Ion Enrichment

Figure 2a illustrates the graphene-NaCl­(aq) interface studied here. We examine pure water and three electrolyte concentrations (0.5, 1, and 2 M NaCl). These concentrations were chosen to highlight ion-induced effects, as previous experiments at the graphene–water interface report negligible changes in the interfacial water response for NaCl concentrations up to 10 mM.^47^ To achieve multinanosecond statistics with near first-principles accuracy, we performed machine learning-based molecular dynamics simulations with a potential trained on revPBE–D3(0) reference data (see Methods section).

**Figure 2:**
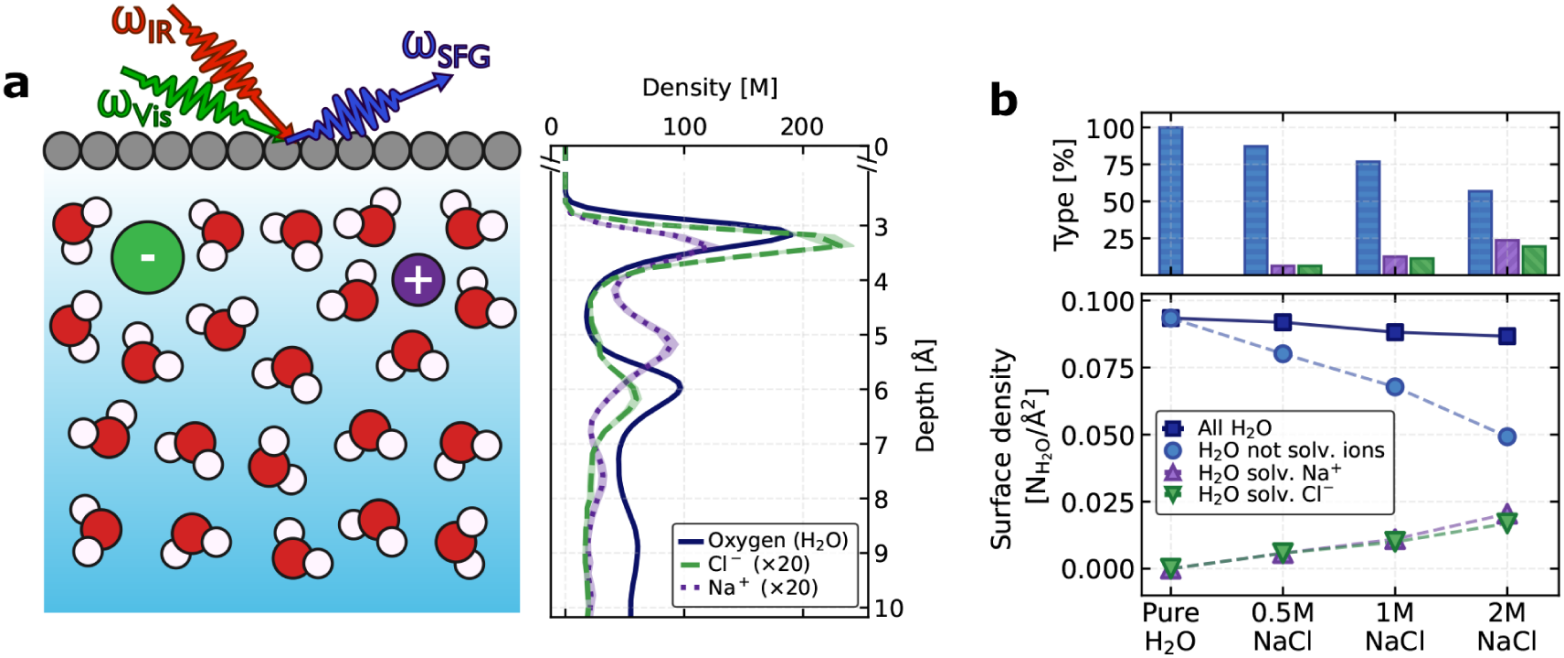
Molecular structure of the graphene–NaCl­(aq) interface at varying concentrations. (a) Schematic illustration of the system studied, along with the density profiles of the water oxygen atoms, Cl^–^ ions, and Na^+^ ions at the graphene–NaCl­(aq) interface for a 2 M NaCl solution. (b) Total number of interfacial water molecules, classified according to whether they solvate no ions, Na^+^, or Cl^–^. The accompanying bar plots show the percentage contribution of each type. Interfacial water molecules were defined as those located between the graphene surface and the first minimum of the water oxygen density profile (see Figure S1), located at approximately 4.5 Å from the surface.

The associated Na^+^ and Cl^–^ ions are schematically depicted near the interface, reflecting the interfacial ion accumulation observed in the density profiles, consistent with our previous work.^57^ The oxygen density profiles exhibit markedly sharper interfacial layering than what is typically seen at the air–water interface,^12,59^ highlighting the stronger structural imprint of the solid substrate.^60,61^ Figure 2b reports the surface density of water molecules in the topmost interfacial layer, classified according to their local environment: those not solvating ions, those solvating Na^+^, and those solvating Cl^–^. With increasing salt concentration, the fraction of ion-solvated water molecules rises (for Cl^–^, from 0% in pure water to 20% at 2 M; for Na^+^, from 0% to 24%). At the same time, the population of non–ion-solvating water decreases (from 100% in pure water to 56% at 2 M), confirming ion adsorption at the interface and the reorganization of hydration shells. Yet the total number of interfacial water molecules remains nearly constant, indicating that the hydrogen-bond network adapts to accommodate ion solvation without significantly disrupting the overall interfacial density and structure.

In the following, unless stated otherwise, we focus on the topmost interfacial water layer, as it provides the dominant contribution to the VSFG spectra, whereas the second layer affects the response only marginally (Figure S4). The thickness of this interfacial region at the graphene–NaCl­(aq) interface, defined as the anisotropic region giving rise to the VSFG signal and quantified as the distance between the graphene surface and the first minimum of the oxygen density profile, is estimated to be ∼4.7 ± 0.1 Å. This value is slightly smaller than the corresponding thickness for pure air–water interface, which has been estimated to be ∼6–8 Å.^62^ Importantly, all structural and spectroscopic trends discussed here are robust with respect to finite-size effects, as verified using systems with both larger cross sections and thicker slit geometries that recover a bulk reference region (Section S5).

Despite the ion enrichment, the computed Im­(χ^(2)^) spectra reveal only subtle changes with increasing salt concentration (Figure 3a). The graphene surface thus appears to buffer the structural impact of interfacial ions. More specifically, upon addition of NaCl, we observe a minor reduction in the magnitude of the hydrogen-bonded O–H band (less negative) and a slight enhancement of the dangling O–H peak (more positive). These changes indicate that ion solvation locally perturbs the orientational structure of nearby interfacial water molecules. Nevertheless, as discussed below, these perturbations are modest, and the population of ion-solvating water molecules remains a minority among the interfacial water molecules. Thus, despite their strong local interfacial enhancement, the resulting changes in the total VSFG response are correspondingly small.

**Figure 3:**
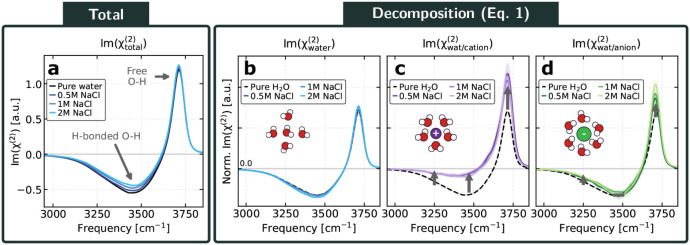
Theoretical VSFG spectra of aqueous NaCl solutions at varying concentrations, with decomposition by O–H bond type. (a) Theoretical Im­(χ^(2)^) spectra for the graphene–NaCl­(aq) interface at NaCl concentrations of 0, 0.5, 1, and 2 M. (b–d) Decomposition of the spectra into contributions from (b) water molecules not solvating ions, (c) Na^+^-solvated waters, and (d) Cl^–^-solvated waters, obtained using the ssVVCF methodology.^58^ The arrows indicate the direction of spectral changes with respect to pure water upon increasing salt concentration, while gray rectangles mark frequency regions where changes are negligible. In panels (b–d), the spectra are normalized by the number of interfacial water molecules of each type.

### Cation Solvation Drives Subtle Changes in the VSFG Spectrum

To gain further insights on why the VSFG spectra exhibit only modest changes with increasing salt concentration, we decompose the response according to the local solvation environment of interfacial water molecules. In this way, $\chi_{\mathrm{total}}^{\left(2\right)}$ can be expressed as a linear combination of three distinct terms. The first term, $\chi_{\mathrm{water}}^{\left(2\right)}$ , arises from water molecules not solvating ions. The second term, $\chi_{\mathrm{wat}/\mathrm{cation}}^{\left(2\right)}$ , comes from water molecules solvating Na^+^. The third arises, $\chi_{\mathrm{wat}/\mathrm{anion}}^{\left(2\right)}$ , from water molecules solvating Cl^–^:^12^

1 $$\chi_{\mathrm{total}}^{\left(2\right)}=\chi_{\mathrm{water}}^{\left(2\right)}+c\left(\chi_{\mathrm{wat}/\mathrm{cation}}^{\left(2\right)}+\chi_{\mathrm{wat}/\mathrm{anion}}^{\left(2\right)}\right)$$

where *c* is a prefactor proportional to the NaCl concentration. The spectra are then normalized by the number of interfacial water molecules in each class, so that we can focus on changes in spectral shape relative to the pure graphene–water interface.

In Figure 3b, we show the spectra from water molecules not solvating ions. We find that their spectral response remains essentially unchanged across all concentrations. This supports our decomposition in eq 1, which can be viewed as a strong-solvation-shell model, where waters outside the ion solvation shells are assumed to remain unperturbed by the presence of salt. We next examine the contribution from Na^+^-solvating water molecules (Figure 3c). Here, the differences are more pronounced. In the hydrogen-bonded region, we observe a strong reduction in the signal magnitude (less negative). In fact, the near-absence of signal suggests that these O–H bonds lie predominantly in-plane. Such orientations do not contribute to the response in the polarization combination used here, and therefore appear inactive in our spectra. In the dangling O–H region, we find an enhanced signal (more positive). These trends indicate that cation solvation locally disrupts the orientational structure of interfacial water. Turning to the Cl^–^-solvating waters (Figure 3d), we see a milder effect. The hydrogen-bonded region remains largely similar to that of pure water, with only a slight reduction in magnitude and no clear dependence on concentration. In the dangling O–H region, there is a moderate increase in signal (smaller than that seen for Na^+^) again with minimal variation across concentrations.

Taken together, these results clarify the origin of the overall spectral changes. The weakening of the hydrogen-bonded O–H band arises primarily from Na^+^-solvating waters, while the enhancement of the dangling O–H signal reflects contributions from both cations and anions, with Na^+^ again playing the dominant role. Crucially, although the spectral response of ion-solvating water molecules differs from that of nonsolvating interfacial water, this difference is small, and such molecules constitute a fraction of the interfacial population (approximately 20% in the 2 M case). As a result, the total $\chi_{\mathrm{total}}^{\left(2\right)}$ spectrum is dominated by water molecules whose structure and orientation remain essentially unchanged, explaining the weak concentration dependence observed in Figure 3a.

### Ion-Specific Coordination Governs Interfacial Water Orientation

We now discuss the origins of the subtle spectral changes. To make these effects clearer, we focus on the 2 M NaCl case, where the differences are most pronounced. We begin by looking at the water molecules that do not solvate ions. Figure 4a shows the two-dimensional (2D) probability distribution of O–H bond orientations as a function of depth from the graphene interface. Each water molecule contributes two O–H bond vectors; an angle of 0° corresponds to O–H bonds pointing into the bulk, whereas 180° corresponds to bonds pointing toward the interface. The distribution shows a dominant feature around 100°, corresponding to O–H bonds lying largely parallel to the surface, and a weaker feature near 25°, associated with a small population of bonds pointing into the bulk. The corresponding 1D projection, shown on the right side of the 2D map, highlights these two features more clearly, with peaks around 25° and 100°, consistent with previous observations of water near hydrophobic surfaces.^63,64^ Most interfacial water molecules therefore adopt in-plane orientations that participate in an extended two-dimensional hydrogen-bond network, while only a minority point out of plane. Because VSFG is sensitive primarily to the out-of-plane component of molecular orientations at the interface, we focus on these less abundant configurations, as they are the ones that determine the spectral differences observed under the particular polarization combination used here. The overall orientation pattern, including both the in-plane network and the SFG-active out-of-plane bonds, is schematically illustrated in Figure 4c. To assess the influence of ions, we compute the difference between this distribution and that for pure water. The difference map confirms that the orientation pattern of water molecules not directly coordinating ions remains largely unchanged (Figure 4b). Only minor deviations are observed, with negative regions indicating weakened features and positive regions indicating emerging ones. Thus, we detect no significant rearrangement beyond the first solvation shell, suggesting that ion effects do not propagate to noncoordinating interfacial water molecules, unlike the longer-range perturbations observed in bulk water.^65^

**Figure 4:**
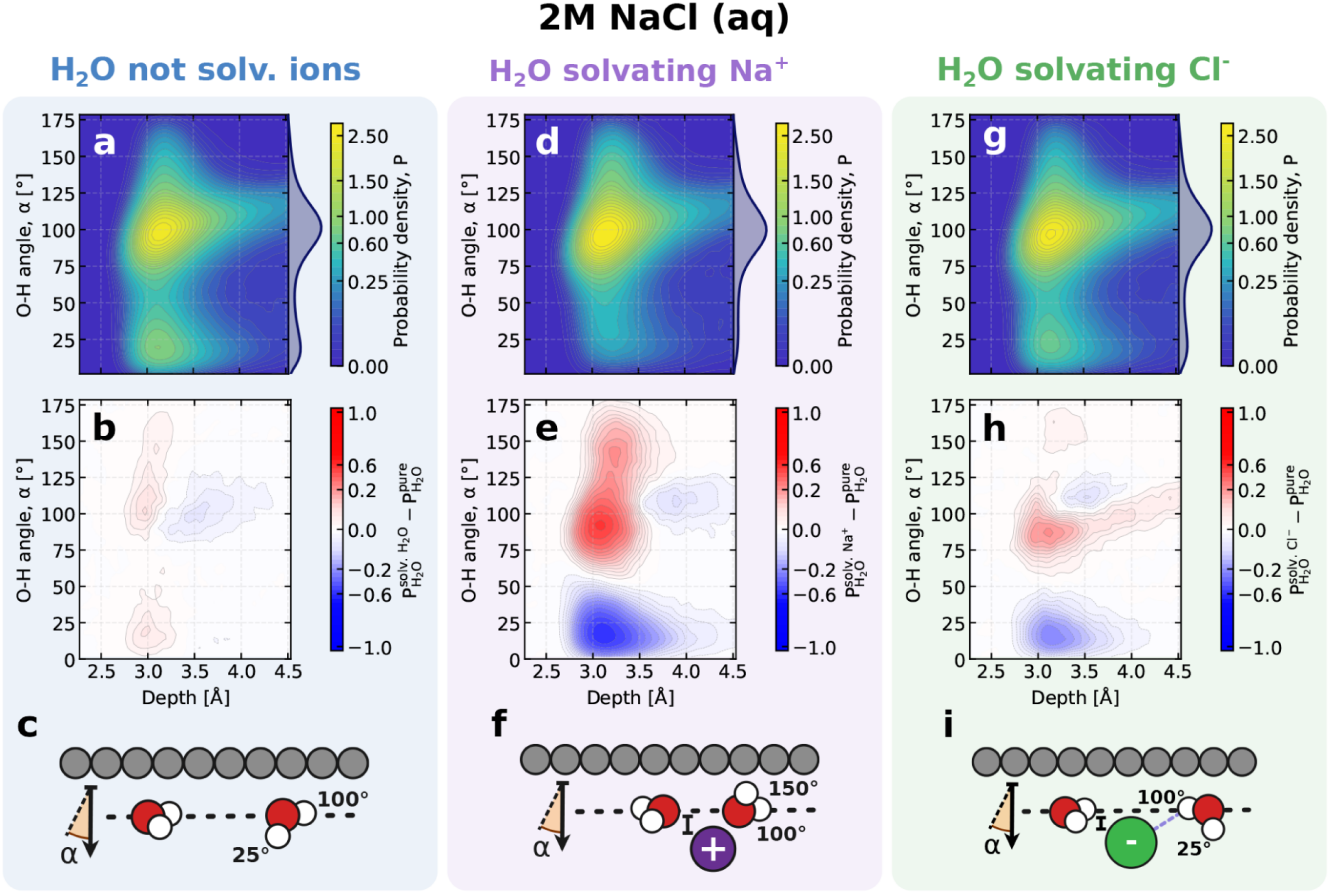
Microscopic analyses of the graphene–NaCl­(aq) interface, with molecular-level illustrations of how Na^+^and Cl^–^ions modify interfacial water structures for a 2 M NaCl solution. (a) Probability distribution of O–H bond orientations in water molecules not solvating ions as a function of their depth, defined as the distance between the water oxygen and the graphene surface, and the angle relative to the surface normal. An angle of 0° indicates that the O–H bond points toward the bulk solution. The accompanying profile shows the O–H angle distribution integrated over all depths, highlighting the most probable orientations. (b) Difference in the probability distribution shown in (a) relative to that of pure water at the graphene interface. Positive values indicate features that appear compared to pure water, while negative values indicate features that disappear compared to pure water. (c) Molecular-level depiction of water molecules solvating other water molecules at the graphene interface, including the definition of the O–H bond angle, α. The horizontal dashed line indicates the position of the interfacial layer based on oxygen atom positions. The left water molecule illustrates the dominant in-plane orientation at the interface, whereas the right one represents the principal out-of-plane configuration. (d–f) Same as (a–c) but for water molecules solvating Na^+^. (g–i) Same as (a–c) but for water molecules solvating Cl^–^. For water molecules surrounding Cl^–^, hydrogen bonding to the ion is indicated by the purple dotted line in (i). Note the relative depths of Na^+^ and Cl^–^ ions with respect to the horizontal dashed line. Results for additional concentrations are provided in Section S2.

We now turn to the analysis of water molecules solvating Na^+^. As shown in the 2D probability distribution of O–H bond orientations in Figure 4d,a single dominant maximum emerges. Comparing this distribution to that of pure water (Figure 4e), we find a pronounced depletion of orientations near 25°, corresponding to the loss of the downward-pointing interfacial configuration. In contrast, there is a modest increase in the probability of larger angles (above 100°). This reduction in hydrogen-bonded O–H orientations, together with the enhanced occurrence of dangling O–H bonds, is consistent with the decrease in the hydrogen-bonded O–H band and the increase in the dangling O–H peak observed in the VSFG spectra (Figure 3c). A schematic representation of water orientations around Na^+^ at the interface is provided in Figure 4f. Importantly, Na^+^ resides slightly below the average interfacial plane defined by the oxygen atoms of the first water layer (Figure 2a). This offset in depth, together with the characteristic dipolar alignment for cations, rationalizes both the observed angular preferences and the corresponding changes in the spectral response.

Lastly, we examine water molecules that solvate Cl^–^. As shown in the 2D probability distribution in Figure 4g, the orientational profile closely resembles that of water molecules not solvating ions, consistent with the minimal spectral changes observed in the VSFG spectra discussed earlier. The corresponding difference map with respect to pure water (Figure 4h) reveals only subtle changes, primarily a depletion of O–H bond orientations around 25°. A schematic of the molecular orientations is shown in Figure 4i. In contrast to the dipolar alignment seen for Na^+^, Cl^–^ solvates water via hydrogen bonding, with one of the hydrogen atoms pointing directly toward the anion. The overall orientation remains broadly similar to that of water molecules not solvating ions, except for this additional hydrogen-bond donor interaction. This O–H bond typically lies nearly parallel to the interface, making it weakly VSFG-active because it lacks a significant out-of-plane projection. Consequently, Cl^–^ ions primarily perturb O–H bonds that are spectroscopically inactive, which explains the relatively modest spectral changes associated with Cl^–^ solvation.

For completeness, we also analyzed the microscopic origins of the subtle variations observed in the free O–H region. This analysis, presented in detail in Section S3, confirms that these weaker spectral features primarily arise from fine adjustments in the orientations of interfacial water molecules.

### Ions Disrupt the Extended Interfacial Hydrogen-Bond Network

So far, we have focused our analysis on the local water structure, characterized by the orientation and hydrogen bonding strength of the O–H bonds. We now take a broader view and examine the topology and connectivity of the interfacial hydrogen-bond network, as well as how it is influenced by the presence of ions. In the absence of salt, interfacial water forms an extended, collective 2D network with hydrogen bonds oriented largely parallel to the surface, consistent with previous observations at the air–water interface^66^ and other weakly interacting (hydrophobic) interfaces^24^ (Figure 5a). Upon the addition of salt, this extended network becomes disrupted, forming fewer rings and exhibiting a slight shift in its distribution toward smaller chains and rings (Figure S13). This restructuring is also reflected in the hydrogen-bond topology: the fraction of DDAA motifs (two donors and two acceptors) decreases with increasing salt concentration. In contrast, DA motifs (one donor and one acceptor) become more prevalent (Figure 5b). At the same time, the average number of hydrogen bonds per water molecule declines with salt concentration, with reductions observed in both intralayer and interlayer bonds (Figure 5c).^67^ Together, these trends reveal a progressive weakening of the extended hydrogen-bond network. Importantly, this restructuring occurs without a significant change in the total number of interfacial water molecules, indicating that ion adsorption reorganizes rather than depletes the interfacial layer, an effect that we have verified to be robust with respect to finite-size effects (see Section S5). The hydrogen-bond network thus adapts to accommodate ions while preserving the overall density and connectivity of interfacial water.

**Figure 5:**
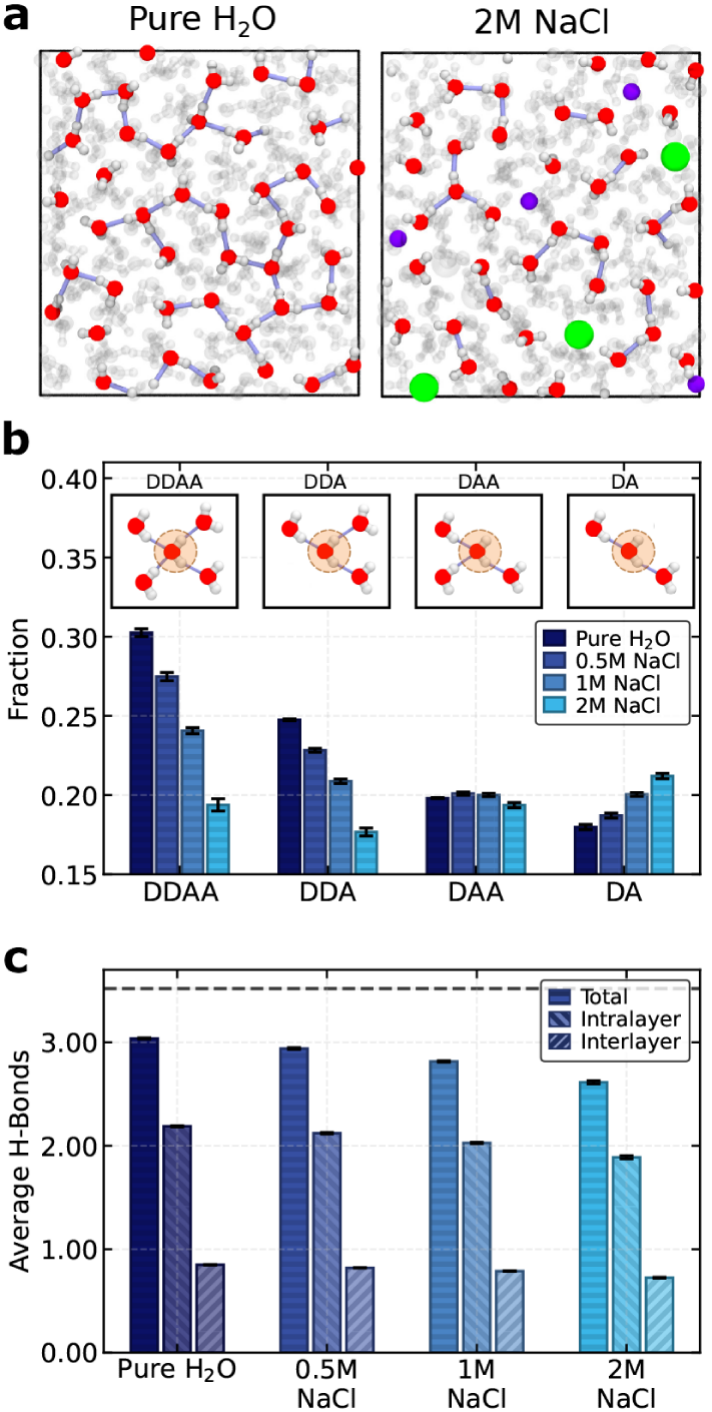
Ion-induced reorganization of the interfacial hydrogen-bond network. (a) Snapshots of the interfacial water structure at the graphene–water interface for pure water and a 2 M NaCl solution. Sodium ions are shown in purple, and chloride ions are in green. (b) Distribution of hydrogen-bond topologies at different NaCl concentrations. Fractions of DDAA, DDA, DAA, and DA motifs are reported, where D and A denote hydrogen-bond donors and acceptors, respectively. The accompanying snapshots illustrate representative examples of the hydrogen-bonding motifs discussed. (c) Average number of hydrogen bonds per interfacial water molecule, decomposed into total, intralayer (within the topmost layer), and interlayer (between the first and second layers). The horizontal dashed blue line marks the bulk-water value.

The restructuring results in a distinct spectroscopic fingerprint, most apparent at low frequencies within the hydrogen-bonded region of the surface-specific H vibrational density of states (Figure S14). In the far-infrared (terahertz, THz) band, at wavenumbers below about 300 cm^–1^, the response is dominated by collective intermolecular motions, including hydrogen-bond translations and librations, as well as ion–water cage modes.^68–70^ The presence of salt redistributes spectral weight within the librational band, depleting cage-like motions (below 80 cm^–1^) while enhancing intermolecular H-bond translations and the librational band (around 400 cm^–1^).^71^ These collective low-frequency modes represent a promising target for future THz-SFG studies,^24,72^ which could directly test how ions reshape the interfacial hydrogen-bond network.

## Discussion

We investigated the organization of ions and the structure of interfacial water at a prototypical hydrophobic interface: the NaCl­(aq)–graphene interface. In this system, ions accumulate strongly at the interface, yet the associated changes in the local structure of interfacial water are relatively modest. This example clearly reveals that the standard paradigm linking ion surface propensity to interfacial water disruption—established for the air–water interface—is not universally applicable to other hydrophobic interfaces and should not be applied uncritically when interpreting experimental results. These differences likely underlie the reported inverse ordering of some molecular ions at graphene interfaces.^34,73,74^

Simulating VSFG spectra is significantly more demanding than simulating bulk spectroscopies, as the signal originates from only a few molecular layers and requires extensive sampling to account for the cancellation of bulk contributions.^58,75,76^ The use of MLPs was therefore instrumental in this work, enabling multinanosecond simulations necessary to resolve the subtle spectral differences underlying our findings—well beyond the reach of direct *ab initio* molecular dynamics. In addition to their computational efficiency, MLPs offer near-first-principles accuracy, which is essential for reliably modeling interfacial properties. Indeed, classical electrostatics predict a universal repulsion of ions from hydrophobic aqueous interfaces,^77,78^ and standard force fields fail to reproduce even the basic enrichment of ions at graphitic surfaces,^57,79,80^ rendering them inadequate for this problem. To avoid interfacial artifacts associated with short-ranged models—such as spurious changes in interfacial water structure and orientation,^81,82^ the MLPs used in this work explicitly incorporate long-range electrostatic interactions, ensuring physical consistency.

Focusing on the microscopic origins of these effects, we find that cations perturb the interfacial water structure more strongly than anions, yet these perturbations remain largely confined to the first solvation shell:^83^ waters not directly coordinating either ion retain their characteristic interfacial arrangement. This short-ranged character of ionic perturbations suggests an enhanced local dielectric screening at the graphene–water interface driven by the graphene polarization. Such screening has been described both by continuum models^84^ and by atomistic simulations,^57^ which show that graphene polarization weakens cation–anion electrostatic attraction and thereby decreases the stability of ion pairs at the interface. The polarizable electronic structure of graphene, arising from its delocalized π electrons, can screen the electric field generated by nearby ions, thereby reducing the stability of contact ion pairs while favoring solvated ionic configurations at the interface. Importantly, first-principles-quality simulations indicate that this effect arises from graphene polarization rather than specific chemical ion–surface bonding.^57^ This picture is further supported by classical and multiscale simulation studies showing that pronounced ion-specific adsorption at graphene–electrolyte interfaces is driven by a combination of ion hydration, surface-induced electrostatic fields, and graphene polarizability.^80,85,86^ As a consequence, the resulting ionic perturbations are highly localized to the interface, in clear contrast to the longer-ranged ionic perturbations typically observed in bulk solutions.^65,87^ We note, however, that these are interfacial effects and should not be mistaken for confinement effects when analyzing graphene-based nanodevices.^88,89^

The charge and rigidity of the interface play pivotal roles. Near the point of zero charge, subtle effects such as those reported here may emerge, whereas at higher charge densities, electrostatic contributions might dominate and potentially overshadow local solvation effects.^90,91^ In this work, we have focused on neutral and flat graphene, which provides a valuable model for nanodevices. Future investigations will extend this approach to charged and flexible surfaces, representing natural next steps, as well as to different ion types. Such studies will help assess the extent to which broader frameworks, such as the Hofmeister series, apply to solid hydrophobic interfaces.

Our results demonstrate that solid–liquid interfaces can host substantial populations of interfacial ions. At 2 M NaCl, roughly half of the interfacial water molecules (about 45%) are solvated by ions, without significantly altering either the number of interfacial water molecules or their local structure. Furthermore, our temperature-dependent analyses reveal that anion adsorption at the graphene–water interface is predominantly entropy-limited, whereas cation adsorption reflects a balance of modest enthalpic and entropic contributions (see Section S6). The ability of carbon-based materials to accommodate dense interfacial ion populations while maintaining a largely unperturbed water structure is particularly beneficial in nanofluidic and electrochemical contexts, where performance depends sensitively on the local organization of nearby water.^92–95^ At the same time, NaCl disrupts the extended two-dimensional hydrogen-bond network, breaking it into smaller domains and weakening the connectivity between adjacent water layers. This reduction in interlayer bonding makes the region behave more hydrophobically, as water becomes less inclined to span across layers.^24^ This modulation could, in turn, be exploited to tune the local acid–base chemistry^96^ and thereby influence electrochemical reactions,^97^ as well as to enhance the solubility of CO_2_ in graphene-based supercapacitors.^98^ More broadly, this duality of local robustness and global lability emerges as a general feature that could be exploited to advance applications in energy storage, conversion, catalysis, and sensing.

## Methods

### Machine-Learning Potential

Molecular dynamics simulations were performed using a committee of eight Behler–Parrinello neural network potentials (NNPs) trained on revPBE-D3(0) reference data^99,100^ developed in our previous work.^57^ This exchange–correlation functional has been shown to describe water–graphene^101,102^ and NaCl–water interactions reliably,^103^ and the resulting NNP reproduces *ab initio* structural and dynamical properties.^57,103^ The short-range interactions were described using atom-centered symmetry functions with a 12 Bohr cutoff, while long-range electrostatics were included via a fixed-charge Coulomb baseline (+1 for Na^+^, −1 for Cl^–^, 0 for C, and SPC/E charges for water). The total energy was expressed as *E* = *E*_sr_ + *E*_Coul_, where the NNP was trained on *E*_sr_ only. Additional details are provided in Section S1.

### Molecular Dynamics Simulations

All simulations were carried out in LAMMPS interfaced with n2p2.^104,105^ Each system consisted of an aqueous NaCl solution confined between two rigid graphene sheets forming a slit pore with lateral dimensions of 19.76 × 21.39 Å^2^ (see Table S1 for further details). Graphene was treated as rigid in the present simulations. Previous work has shown that introducing graphene flexibility has little effect on interfacial ion solvation and adsorption free energies at the graphene–water interface.^20^ Periodic boundary conditions were applied in all directions, and a vacuum layer three times the slit height was included along *z* to remove spurious interactions. Electrostatics were computed using the PPPM method, and the Yeh–Berkowitz correction^106^ was applied to account for the slab geometry. After 25 ps of NVT equilibration, production simulations of 200 ps were performed in the NVE ensemble, averaged over 40 independent trajectories, totaling over 30 ns of sampling. Temperature was maintained at 300 K using a Nosé–Hoover thermostat with a 0.5 fs time step. Graphene atoms were fixed during production runs.

### Data Analysis

VSFG spectra were computed via the surface-specific velocity–velocity correlation function (ssVVCF) approach.^58^ The analysis focused on the topmost interfacial water layer, which dominates the spectral response (Figure S4). We verified that moderate variations in these cutoffs do not qualitatively affect our conclusions. The hydrogen bonds are identified using the geometric definition provided in ref 107. Interfacial water molecules are defined as those located between the graphene surface and the first minimum of the water oxygen density profile (see Figure S1), corresponding to a distance of approximately 4.5 Å from the surface. Additional details are provided in Section S1.

### Sample Preparation and HD-VSFG Measurement

The preparation of suspended graphene on the water surface followed procedures similar to those reported previously.^47,52,73^ Details have been presented in ref 47. HD-SFG measurements were performed using a noncollinear setup driven by a Ti:sapphire regenerative amplifier laser system (800 nm central wavelength, 40 fs pulse width, 5 mJ pulse energy, 1 kHz repetition rate). The configuration of the optical setup has been described previously.^47,108^ For heterodyne detection, the local oscillator (LO) was generated by focusing the IR and visible beams onto a 200 nm-thick ZnO film deposited on a 1 mm-thick CaF_2_ substrate, following established procedures.^109^ The LO, IR, and visible beams were subsequently directed and refocused using pairs of off-axis parabolic mirrors to achieve spatial and temporal overlap at the graphene–water interface. The incidence angles (in air) for the IR, visible, and LO beams were 50°, 61°, and 64°, respectively. Measurements were carried out under the *ssp* polarization combination (*s*-polarized SFG and visible beams, *p*-polarized IR) in a dry air atmosphere to minimize interference from water vapor. PPP measurements were also explored but are not reported here, as the response in this polarization is dominated by graphene rather than interfacial water, consistent with previous studies.^110–112^ Phase referencing was performed using z-cut quartz, and the sample height was checked with a displacement sensor (CL-3000, Keyence).

## Supplementary Material



## Data Availability

All data required to reproduce the findings of this work are openly available on GitHub (https://github.com/water-ice-group/sfg-gra-water-nacl).

## Notes

Additional simulation details, including methods and system setup; further system characterization; detailed analysis of dangling O–H groups; hydrogen-bond network topology and connectivity; assessment of finite-size effects; and temperature-dependent thermodynamics of ion adsorption at the graphene–water interface (PDF)
