# Supplementary material for: Breaking the Air–Water Paradigm: Ion Behavior at Hydrophobic Solid–Water Interfaces
Source: J Am Chem Soc. 2026 Mar 19;148(12):12753–63. doi: 10.1021/jacs.5c20209 (PMC13047698; doi:10.1021/jacs.5c20209)
Supplement: Supplementary file 1 [file ja5c20209_si_001.pdf]

Supporting Information for: Breaking the Air–Water Paradigm: Ion Behavior at Hydrophobic Solid–Water Interfaces

Xavier R. Advincula,[1,2,3] Kara D. Fong,[4,5] Yongkang Wang,[6] Christoph Schran,[2,3] Mischa Bonn,[6] Angelos Michaelides,[1,3,a)] and Yair Litman[6,b)]

[1)] *Yusuf Hamied Department of Chemistry, University of Cambridge, Lensfield Road, Cambridge, CB2 1EW, UK*

[2)] *Cavendish Laboratory, Department of Physics, University of Cambridge, Cambridge, CB3 0HE, UK*

[3)] *Lennard-Jones Centre, University of Cambridge, Trinity Ln, Cambridge, CB2 1TN, UK*

[4)] *Division of Chemistry and Chemical Engineering, California Institute of Technology, Pasadena CA 91125, USA*

[5)] *Marcus Center for Theoretical Chemistry, California Institute of Technology, Pasadena CA 91125, USA*

[6)] *Max Planck Institute for Polymer Research, Ackermannweg 10, 55128 Mainz, Germany*

[a)] Electronic mail: am452@cam.ac.uk

[b)] Electronic mail: litmany@mpip-mainz.mpg.de

CONTENTS

S1. Additional simulation details S3

Methods S3

System Setup S6

S2. Additional system characterization S10

S3. Dangling O–H characterization S14

Ion Coordination Enhances Dangling O–H Orientation at the Interface S15

S4. Hydrogen-bond network topology and connectivity S19

S5. Assessment of Finite-Size Effects S21

S6. Temperature-Dependent Thermodynamics of Ion Adsorption at the Graphene–Water Interface S23

References S26

S1. ADDITIONAL SIMULATION DETAILS

Methods

Machine Learning Potential. In this work, molecular dynamics simulations are performed using a committee of eight Behler–Parrinello neural network potentials (NNPs) developed in our previous work.[S1] The NNPs were trained on reference data from revPBE-D3(0) density functional theory[S2,S3] capturing both bulk and nanoconfined aqueous NaCl configurations across varying concentrations and confinement widths. This has been shown to describe water–graphene interactions reliably[S4,S5] as well as NaCl–water interaction.[S6]

This training set explicitly includes interfacial environments similar to those studied here, such as the NaCl(aq)–graphene interface, allowing for the accurate modeling of ion–water and ion–surface interactions at the solid–liquid boundary. To efficiently describe interatomic interactions, the models use atom-centered symmetry functions with a 12 Bohr cutoff, accounting for short-range interactions. Long-range electrostatics are treated separately using a fixed-charge Coulomb baseline: +1 for cations, -1 for anions, 0 for carbon, and SPC/E charges for water. The total system energy is partitioned as $E = E_{\mathrm{sr}} + E_{\mathrm{Coul}}$, where the NNP is trained only on the short-range component E_{sr} and its associated forces. During simulations, the full energy and forces are obtained by combining the NNP and Coulomb contributions.

We thoroughly validated the NNP against *ab initio* molecular dynamics, showing excellent agreement in key structural and dynamical properties, including radial distribution functions, density profiles, and vibrational spectra.[S1] In addition, autocorrelation functions and computed bulk electrolyte conductivities align closely with experimental observations,[S6] further confirming the model's reliability across relevant observables.

Molecular Dynamics Simulations. Molecular dynamics simulations were performed using the LAMMPS interface to the n2p2 NNP framework.[S7,S8] All systems were constructed in orthorhombic simulation cells with periodic boundary conditions in all directions. Each system consists of an aqueous NaCl solution confined between two parallel, rigid graphene sheets that form a slit pore. The slit pore is wide enough to sustain bulk-like water density at its center (see Figure S1). This setup enables the study of well-defined solid–liquid interfaces on both sides of the channel, facilitates statistical convergence, and allows direct comparison

with previous slit-pore simulations.[S1] To eliminate spurious interactions along the z-axis (perpendicular to the interfaces), a vacuum layer at least three times the slit height was introduced, and the Yeh–Berkowitz correction[S9] was applied to account for the slab geometry properly. Long-range electrostatics were treated using the particle–particle particle-mesh (PPPM) method. Initial configurations were generated by randomly packing water molecules and NaCl ions between graphene sheets, followed by one ns of NVT equilibration with classical force fields, using the SPC/E water model, Dang ion parameters,[S10] and Werder carbon–water interactions.[S11] This was followed by NVT equilibration of 25 ps using the NNP. The equations of motion were integrated using a velocity-Verlet algorithm with a 0.5 fs time step, and temperature was maintained at 300 K via a Nosé–Hoover thermostat (damping time: 50 fs). Production simulations were carried out in the NVE ensemble. For each state point, observables were averaged over 40 independent 200 ps trajectories to ensure robust statistics. The reported error bars reflect the standard error across these replicas. Across the four concentrations examined (pure water, 0.5, 1, and 2 M NaCl), this amounts to a cumulative sampling time exceeding 30 ns. Forces were evaluated as the average over a committee of eight independently trained NNPs, ensuring robust statistical sampling. We note that nuclear quantum effects were not included, as they are not expected to alter the observed trends.[S12] However, they may influence the results semi-quantitatively, particularly with respect to the lifetime of dangling O–H bonds[S13,S14]

The NaCl-to-water ratios used in this work were approximately 1:110, 1:55, and 1:27.5, corresponding to bulk concentrations of 0.5 M, 1 M, and 2 M, respectively. Each graphene sheet was constructed with lateral dimensions of 19.760 Å by 21.390 Å. To determine the appropriate slit height, and thereby the solution density, we performed additional equilibration runs in which one graphene layer was allowed to move along the z-axis under the influence of a piston exerting a pressure of 1 atm. While the number of water molecules was kept fixed across all concentrations, the resulting slit heights varied slightly to accommodate the different salt contents. The precise dimensions and number of molecules in each system are summarized in Table S1. We emphasize that although graphene sheets were mobile in the piston simulations, all carbon atoms were kept fixed during the production runs.

Data Analysis. The VSFG spectra from molecular dynamics simulations were computed using the surface-specific velocity–velocity correlation function (ssVVCF) approach,[S15] applying a Hahn window of 1 ps. The spectra represent an average over 40 independent 200-ps

trajectories performed in the NVE ensemble, each initiated from uncorrelated configurations extracted from NVT simulations. Throughout this work, we focus on the topmost interfacial water layer, which we show in Figure S4 to be the primary contributor to the VSFG response. Including deeper layers, such as the second water layer, has minimal effect on the spectral features. A central part of our analysis involves decomposing the interfacial response into contributions from three distinct water populations: H_2O-coordinated waters (not coordinating any ions), Na^+-coordinated waters, and Cl^--coordinated waters. A water molecule is considered coordinated to Na^+ or Cl^- if its oxygen atom lies within 3.4 Å or 4.0 Å of the respective ion. If a water molecule satisfies both criteria simultaneously, it is counted as coordinated to both ions. These distance thresholds correspond to the first minima of the respective radial distribution functions. We verified that moderate variations in these cutoffs do not qualitatively affect our conclusions. Finally, to quantify the presence of dangling O–H bonds across different systems, we adopted the definition from Ref. S16. In this framework, an interfacial O–H bond is considered as dangling if the distance between its oxygen atom and any other water oxygen ($O\cdots O$ distance) exceeds 3.5 Å or the $H–O\cdots O$ angle is greater than 50°. Otherwise, the O–H bond is classified as hydrogen-bonded. To compute the fraction of dangling O–H bonds, we follow previous studies[S16–S19] and consider the sum of DA and DAA populations, where D and A represent the number of hydrogen-bond donors and acceptors per water molecule, respectively.

System Setup

We modeled aqueous NaCl solutions confined between two parallel rigid graphene sheets (19.760 Å by 21.390 Å), forming slit pores wide enough to sustain bulk-like water density at the center (see Figure S1). Salt-to-water ratios of approximately 1:110, 1:55, and 1:27.5 correspond to bulk concentrations of 0.5, 1, and 2 M, respectively. The number of water molecules was kept constant across concentrations, with the slit height adjusted through piston equilibration runs, where one graphene sheet was allowed to move along the z-axis under 1 atm pressure. This procedure yielded slightly different pore widths depending on the salt content. Final system dimensions and compositions are reported in Table S1. During production simulations, all graphene atoms were held fixed.

TABLE S1. Overview of the systems considered in this work. For each system, we report their slit width, W; the graphene dimensions; the number of water molecules, $N_{\mathrm{H_2O}}$; the number of Na^+ ions, $N_{\mathrm{Na^+}}$; the number of Cl^- ions, $N_{\mathrm{Cl^-}}$; the concentration; the number of independent runs, N_{runs}; and the simulation production time, t_{sim}.

W	Graphene dims. [Å×Å]	$N_{\mathrm{H_2O}}$	$N_{\mathrm{Na^+}}$	$N_{\mathrm{Cl^-}}$	Conc. [M]	N_{runs}	t_{sim} [ps]
19.36	19.760 × 21.390	232	0	0	0	40	200
19.61	19.760 × 21.390	232	2	2	0.5	40	200
19.85	19.760 × 21.390	232	4	4	1	40	200
20.35	19.760 × 21.390	232	8	8	2	40	200

The corresponding water density profiles across the slit pores are shown in Figure S1. As illustrated in Figure S1b, the interfacial water molecules were defined as those located between the graphene surface and the first minimum of the water oxygen density profile, found at approximately 4.5 Å from the surface.

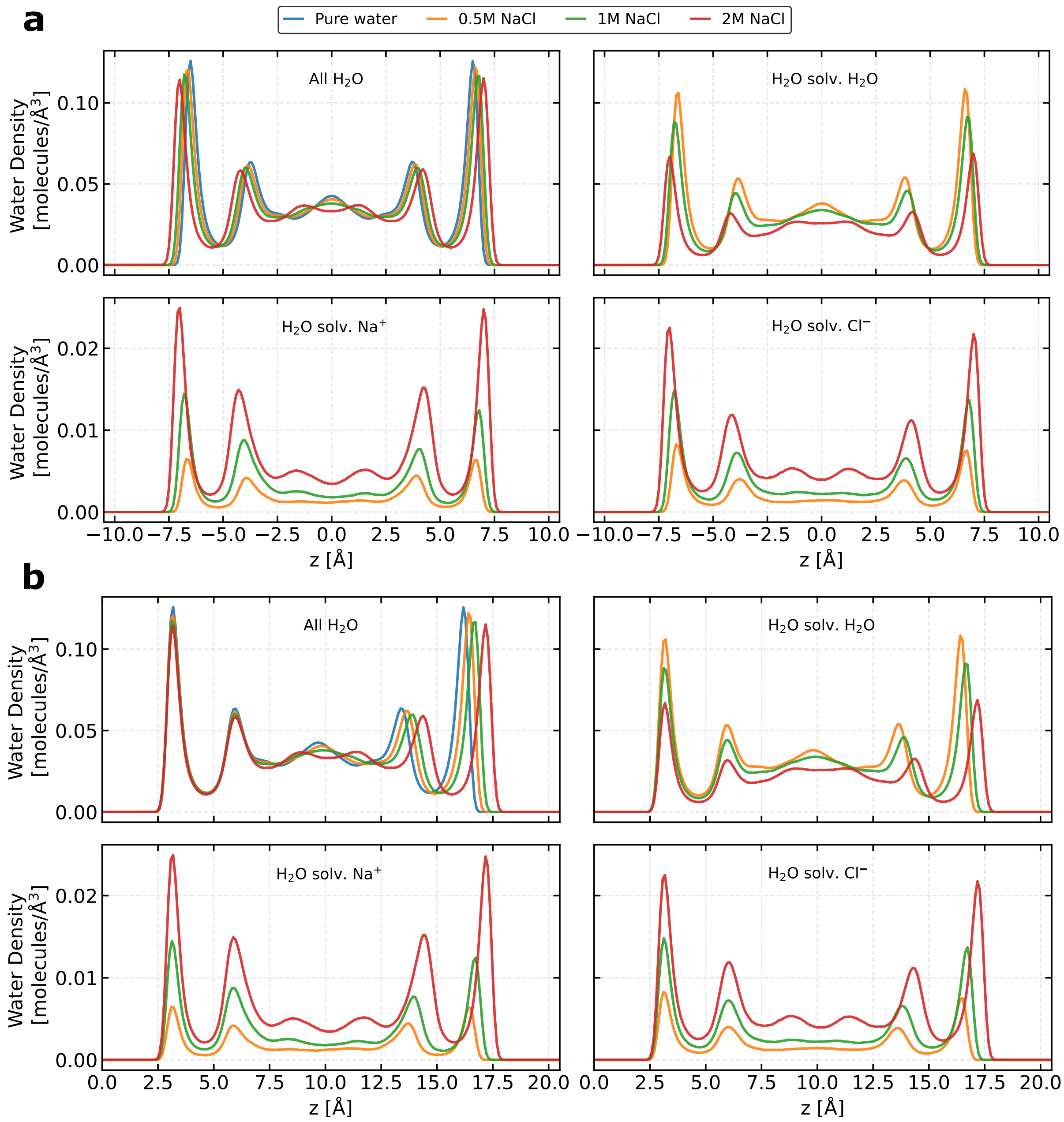

FIG. S1. Density profiles of water across the slit pore, shown (a) symmetrized about $z = 0$ and (b) referenced to a single graphene interface. Water molecules are classified into four categories: all waters, waters solvating other waters (not ions), waters solvating Na^+, and waters solvating Cl^-.

For completeness, in Figure S2 we show the total water density profile accompanied by the ion density.

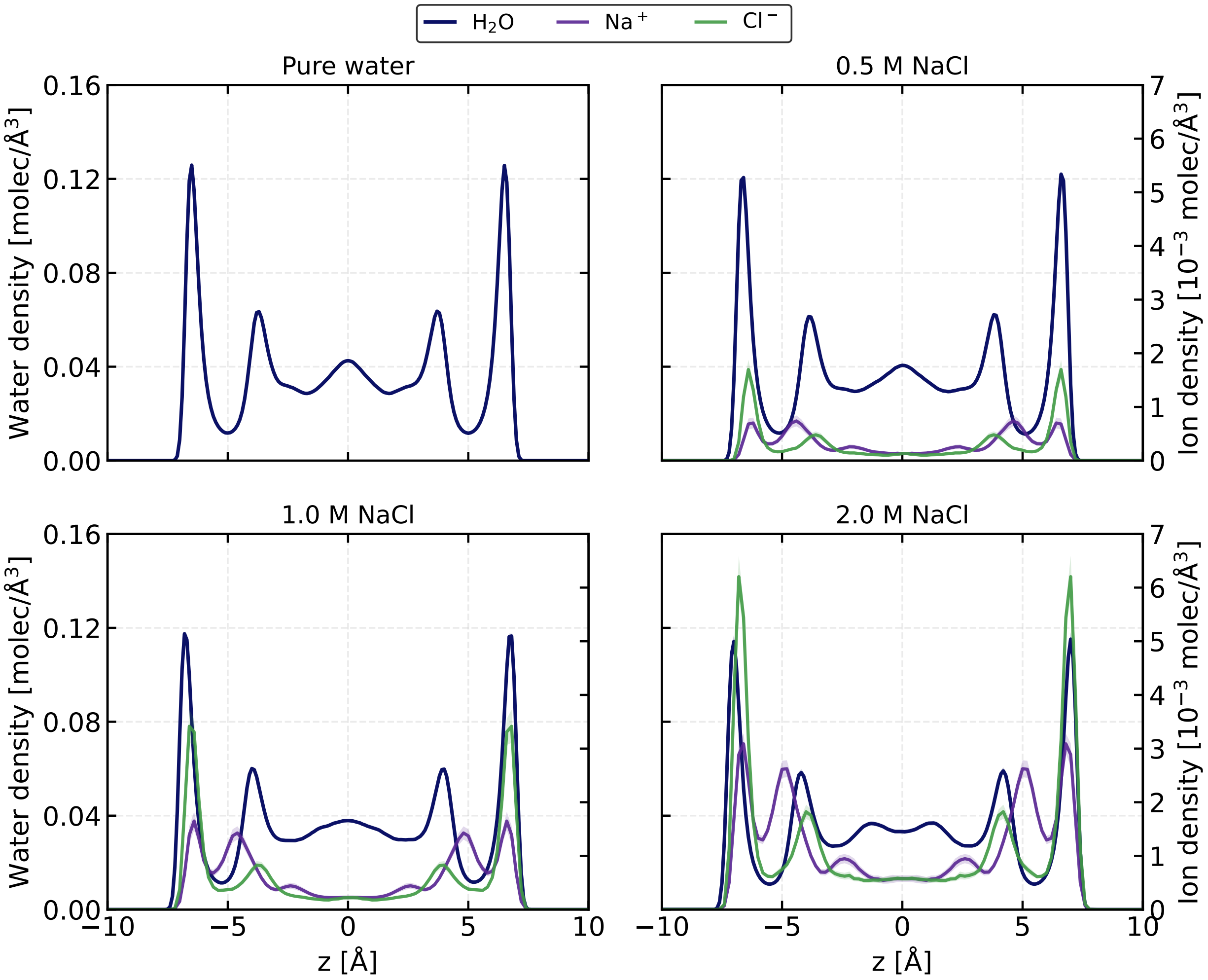

FIG. S2. Symmetrized density profiles of water and ions across the slit pore for the different NaCl concentrations studied. The total water density is shown together with the corresponding Na^+ and Cl^- ion densities, all symmetrized about $z = 0$.

Representative snapshots of the NaCl solutions considered in this work are shown in Figure S3.

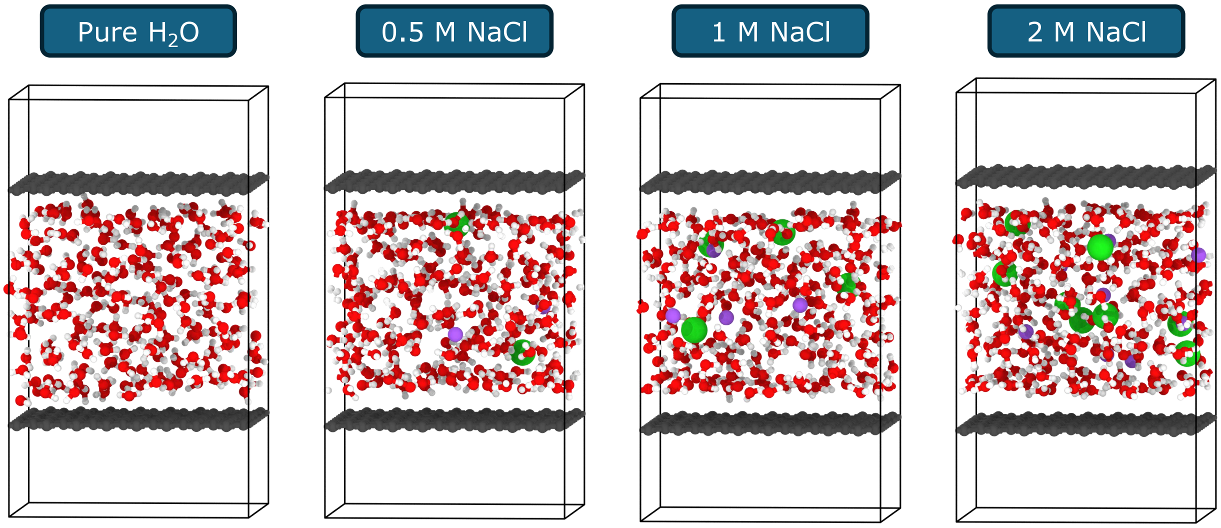

FIG. S3. Representative simulation snapshots of the slit-pore systems studied.

S2. ADDITIONAL SYSTEM CHARACTERIZATION

In the main text, we focus on the topmost interfacial water layer, which provides the dominant contribution to the spectra, while the second layer influences the response only marginally. Figure S4 illustrates this by comparing VSFG spectra computed from only the topmost layer with those including the first two layers.

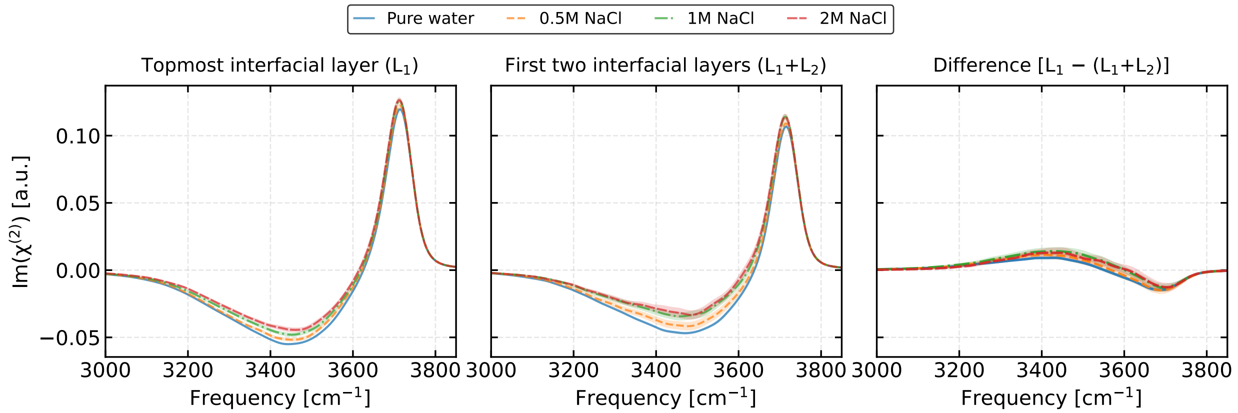

FIG. S4. VSFG spectra of interfacial water at the graphene–water interface. (Left) Contribution from the topmost interfacial layer, L_1. (Center) Contribution from the first two interfacial layers, L_1+L_2). (Right) Difference spectrum, highlighting the marginal influence of the second layer on the overall response. Results are shown for pure water and NaCl solutions at concentrations of 0.5 M, 1 M, and 2 M.

In Figure S5, we present the orientational profiles of the different water classes, along with the same profiles weighted by their density. These weighted profiles act as a proxy for understanding VSFG variations and show agreement with the observations reported in the main text.

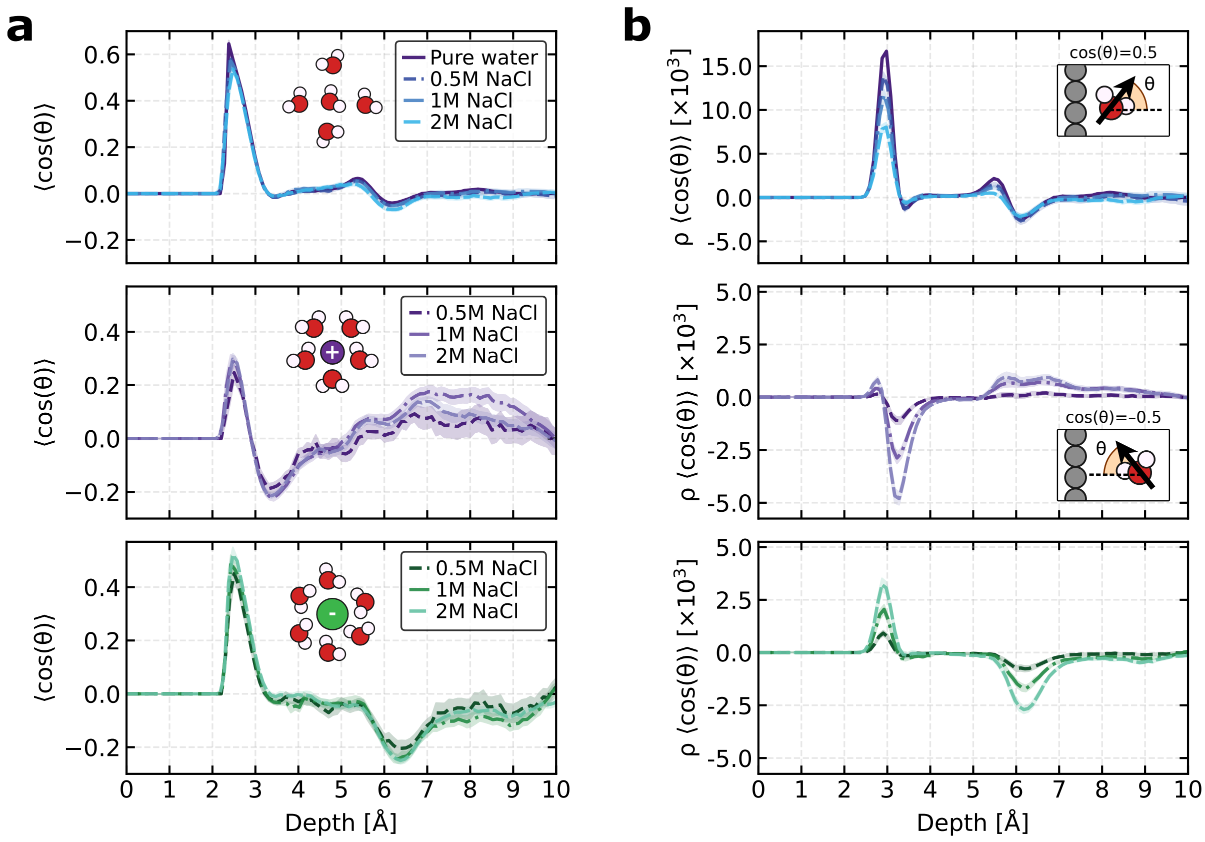

FIG. S5. (a) Average orientation of the H–O–H bisector of water molecules relative to the surface normal, and (b) product of the water density profiles and the average orientation. Results are shown separately for free water molecules (blue shades), water molecules coordinating Na^+ ions (purple shades), and those coordinating Cl^- ions (green shades). A positive (negative) $\langle \cos(\theta) \rangle$ value indicates net orientation towards the bulk (towards the graphene surface).

In Figures S6 and S7, we present microscopic analyses of the graphene–NaCl(aq) interface for 0.5 and 1 M solutions, complementing the 2 M case discussed in the main text.

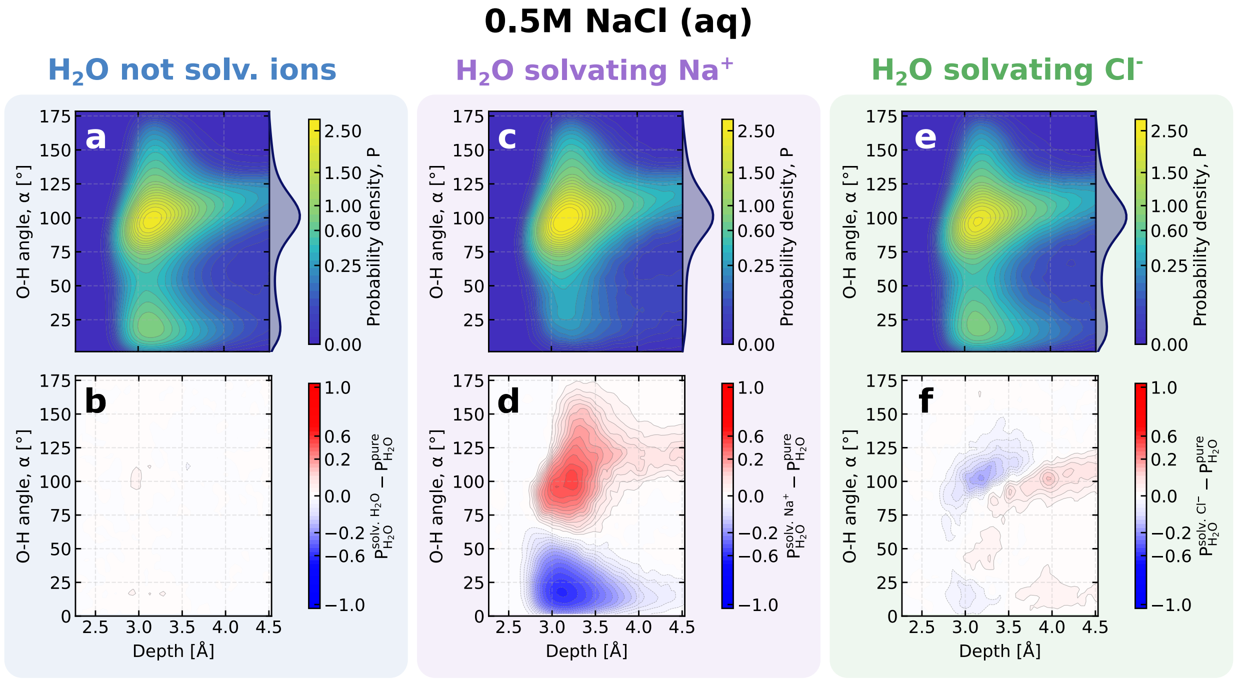

FIG. S6. Microscopic analyses of the graphene–NaCl(aq) interface for a 0.5 M NaCl solution. (a) Probability distribution of O–H bond orientations in water molecules not solvating ions as a function of their depth from the graphene interface and their angle relative to the surface normal. (b) Difference in the probability distribution shown in (a) relative to that of pure water at the graphene interface. Positive values indicate features that appear compared to pure water, while negative values indicate features that disappear compared to pure water. (c–d) Same as (a–b) but for water molecules solvating Na^{+}. (e–f) Same as (a–b) but for water molecules solvating Cl^{-}.

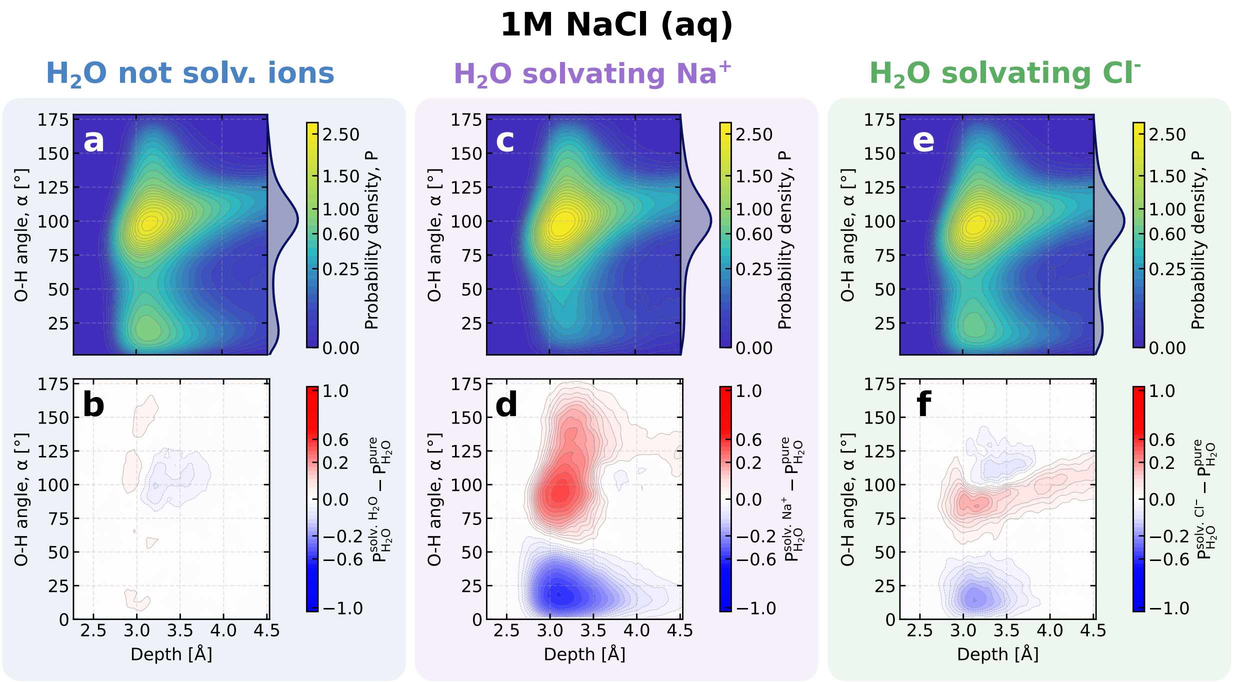

FIG. S7. Microscopic analyses of the graphene–NaCl(aq) interface for a 1 M NaCl solution. (a) Probability distribution of O–H bond orientations in water molecules not solvating ions as a function of their depth from the graphene interface and their angle relative to the surface normal. (b) Difference in the probability distribution shown in (a) relative to that of pure water at the graphene interface. Positive values indicate features that appear compared to pure water, while negative values indicate features that disappear compared to pure water. (c–d) Same as (a–b) but for water molecules solvating Na^+. (e–f) Same as (a–b) but for water molecules solvating Cl^-.

S3. DANGLING O–H CHARACTERIZATION

An interfacial O–H bond is classified as 'free' if the distance between its oxygen atom and any other water oxygen (O$\cdots$O) exceeds 3.5 Å and the D–O$\cdots$O angle is greater than 50°; otherwise, it is considered hydrogen-bonded. This definition follows Ref. S16. The fraction of H_2O molecules carrying free O–H bonds is obtained by summing the DA and DAA populations, where D and A denote the number of hydrogen-bond donors and acceptors per water molecule, respectively. This analysis is restricted to the interfacial region, defined as

$$z \leq -z_G + 1.8\ \text{Å} \quad \text{or} \quad z \geq +z_G - 1.8\ \text{Å},$$

with z_G the z-coordinate of the Gibbs dividing surface. The definition of the interfacial region and the position of z_G are illustrated in Figure S8.

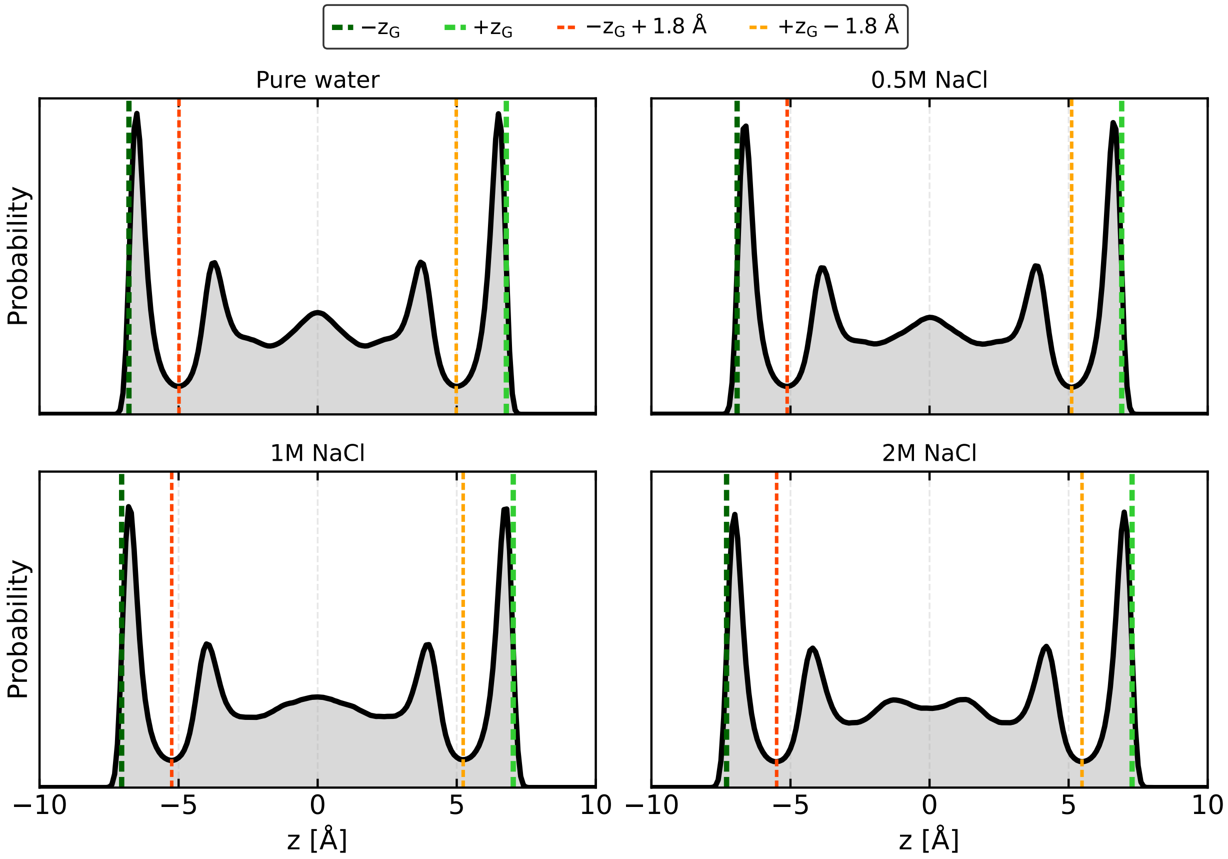

FIG. S8. Density profiles across the slit pore along the surface normal (z) for pure water and NaCl solutions at different concentrations. The Gibbs dividing surfaces ($\pm z_G$) and the boundaries used to define the interfacial region ($-z_G + 1.8$ Å and $+z_G - 1.8$ Å, orange lines).

Ion Coordination Enhances Dangling O–H Orientation at the Interface

Here we examine how salt influences the population of dangling O–H bonds at the graphene–water interface. To identify such species, as previously introduced, we adopt the well-established definition of dangling O–H bonds based on the absence of hydrogen bonding interactions, following the criteria of Ref. S16. While the 2D orientation–depth maps in Figure 4 provide rich structural insight, they are less informative for detecting dangling O–H bonds due to their relatively low abundance, as evidenced in Figure S9.

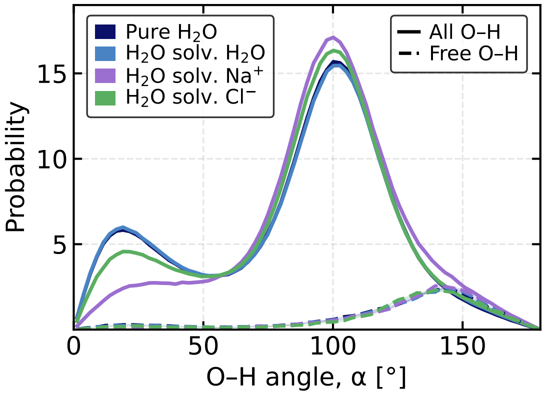

FIG. S9. Orientational distributions of all interfacial O–H bonds (solid lines) and free O–H bonds only (dashed lines) for pure water, H_2O-solvating waters, Na^+-solvating waters, and Cl^--solvating waters in a 2 M NaCl solution.

To quantify how salt and its concentration affect interfacial structure, we calculate the fraction of interfacial O–H bonds considered to be dangling bonds within each water environment. Figure S10 shows this dangling O–H fraction as a function of concentration, resolved by water type.

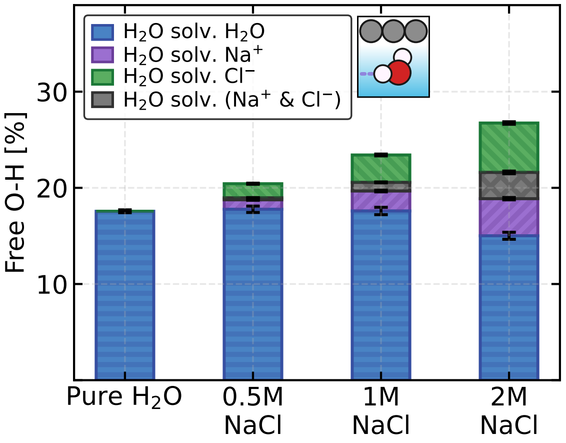

FIG. S10. **Dangling O–H bond characterization at the graphene–NaCl(aq) interface.** Fraction of dangling O–H bonds among interfacial (first-layer) water molecules as a function of NaCl concentration, separated into contributions from H_2O-solvating waters, Na^+-solvating waters (not coordinating Cl^-), Cl^--solvating waters (not coordinating Na^+), and waters simultaneously coordinating both ions. The schematic illustrates a dangling O–H bond of an interfacial water molecule and a hydrogen-bonded O–H bond, which give rise to distinct signatures in the VSFG spectra.

As it can be seen in Figure S10, across all concentrations we observe an overall increase in the proportion of dangling O–H bonds upon salt addition. When normalized with respect to the total number of interfacial water molecules, water solvating Cl^- appears to contribute a comparable or even larger fraction than those solvating Na^+. This reflects the larger hydration shell of Cl^-,[S6] meaning that each anion coordinates more interfacial waters, rather than a higher propensity of individual Cl^--bound waters to expose dangling O–H bonds (see Figure S11).

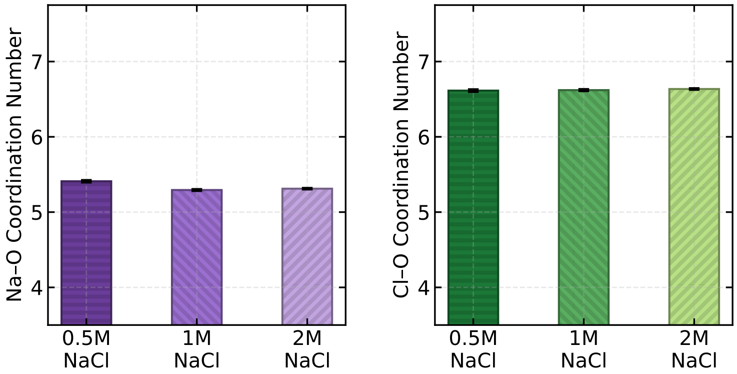

FIG. S11. Average Na–O and Cl–O coordination numbers at different NaCl concentrations (0.5 M, 1 M, and 2 M).

When the data are instead normalized by water type (Figure 4), Na^+-coordinated waters indeed exhibit a higher dangling O–H fraction. This trend parallels the enhancement of the dangling O–H peak in the VSFG spectra (Figure 3b,c), confirming that the spectral changes reflect genuine structural differences at the interface. Notably, in 1 M and 2 M solutions, the dangling O–H population at the graphene–electrolyte interface is higher than that reported for the air–water interface (about 24%[S17]). To probe this effect further, we compute the lifetimes of dangling O–H bonds, which are experimentally accessible via pump–probe VSFG spectroscopy.[S20] For this, we calculated the lifetime of free O–H bonds using the time correlation function

$$C(t) = \frac{\langle n(0)\, n(t) \rangle}{\langle n(0) \rangle},$$

where $n(t) = 1$ if an O–H bond is classified as free at time t, and $n(t) = 0$ otherwise. The term $\langle n \rangle$ denotes the ensemble average of n. We can now fit $C(t)$ with a double exponential of the form,[S21]

$$C(t) = a\, e^{-\frac{t}{\tau_f}} + b\, e^{-\frac{t}{\tau_s}} + c,$$

where a, b, and c are fitting coefficients. The time constants τ_f and τ_s represent the fast and slow components, respectively. τ_f reflects the librational motion of water, while τ_s corresponds to the reorientation of the free O–H bond as it rotates and eventually forms a hydrogen bond with another water molecule at the interface. As shown in Figure S12, these lifetimes increase with salt concentration.

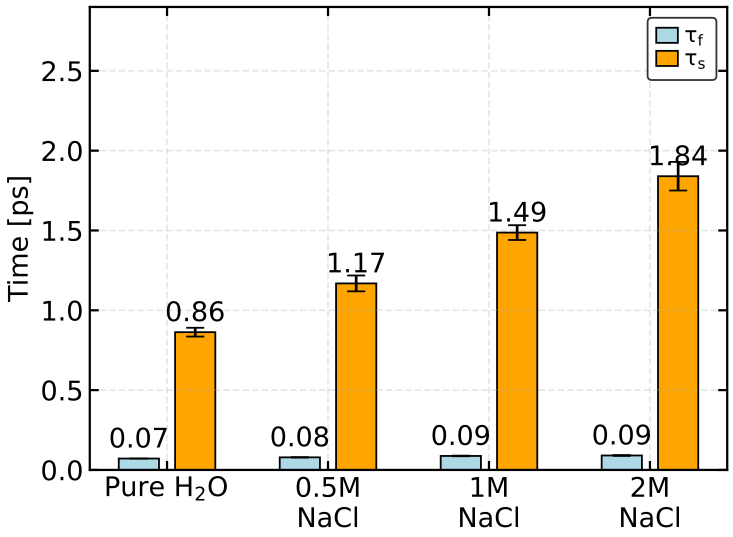

FIG. S12. Lifetimes of free O–H bonds at the graphene–water interface as a function of NaCl concentration. Bars show the fitted fast (τ_f) and slow (τ_s) components obtained from biexponential fits of the correlation function $C(t)$. Error bars denote the fitting uncertainties.

At the air–water interface, the lifetime is about 1.06 ps,[S17] whereas at the graphene–water interface studied here, it ranges from 0.86 ps in pure water to 1.84 ps at 2 M NaCl, highlighting the distinct dynamical response of dangling O–H bonds at solid–water interfaces. As spectral shapes alone can be ambiguous, lifetime measurements provide a readily accessible experimental signature and more robust route to disentangling interfacial structural and dynamical effects, thereby establishing a benchmark for future experimental validation.

S4. HYDROGEN-BOND NETWORK TOPOLOGY AND CONNECTIVITY

In the main text, we described how salt disrupts the extended hydrogen-bond network at the graphene–water interface, breaking large two-dimensional structures. Figure S13a confirms that, upon the addition of salt, fewer rings are formed. Figure S13b further shows a subtle shift in the distribution toward smaller chains and rings.

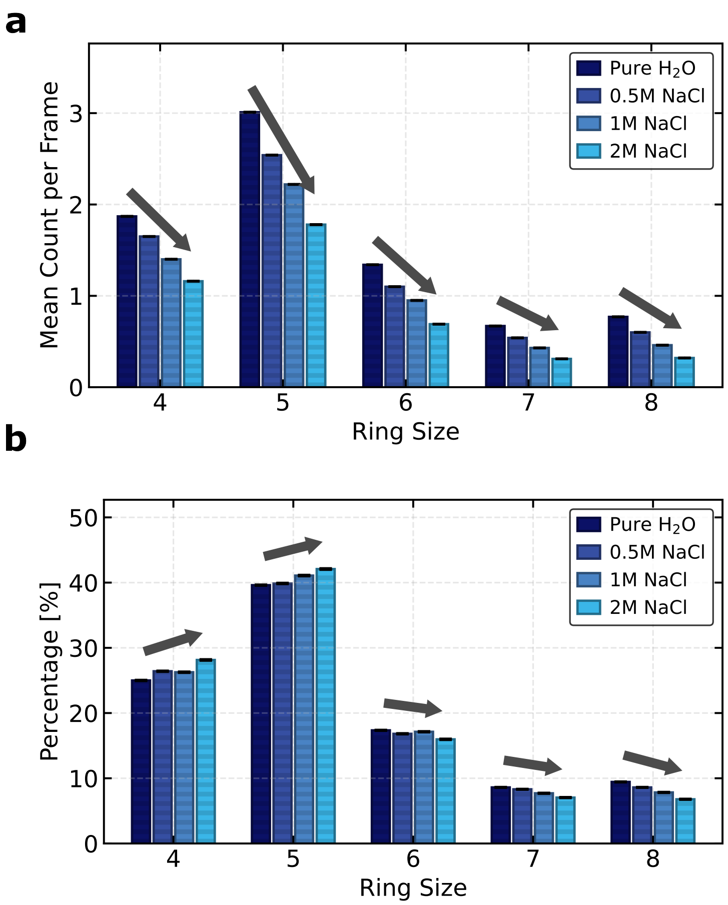

FIG. S13. (a) Mean count per frame and (b) distribution of hydrogen-bonded ring sizes in interfacial water for pure water and NaCl solutions at 0.5 M, 1 M, and 2 M.

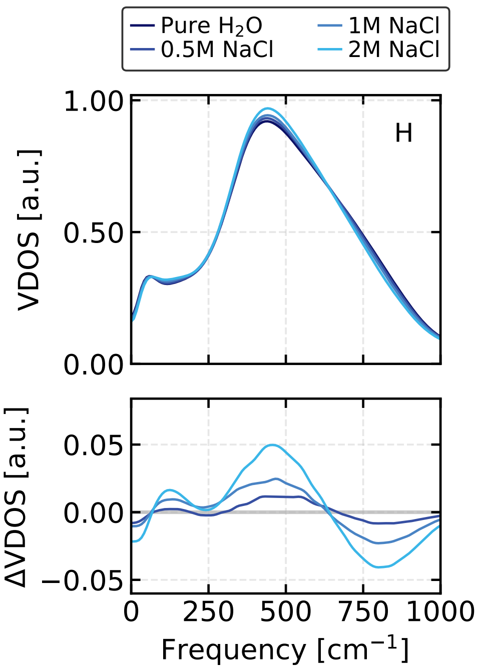

FIG. S14. Low-frequency interfacial vibrational density of states (VDOS) for H as a function of NaCl concentration, with ΔVDOS shown relative to pure H_2O.

S5. ASSESSMENT OF FINITE-SIZE EFFECTS

In the main text, simulations were performed using graphene sheets with lateral dimensions of 19.760 Å × 21.390 Å. To assess the possible influence of finite-size effects, we carried out additional simulations using a larger simulation cell with lateral dimensions of 29.640 Å × 29.946 Å. As shown in Figure S15, increasing the system size has no discernible impact on the structural properties of interfacial water, including the 2D in-plane hydrogen-bond network, nor on the calculated VSFG spectra. This demonstrates that finite-size effects are negligible for the observables considered in this work, and confirms the robustness of the conclusions drawn in the main text.

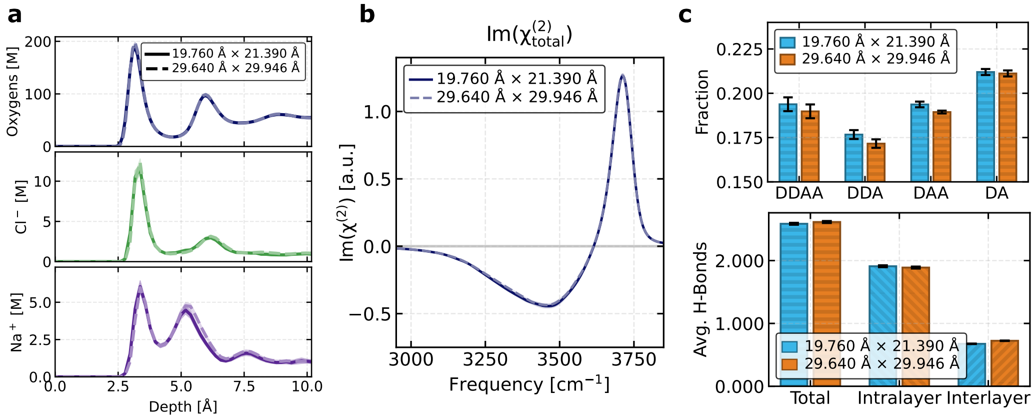

FIG. S15. Evaluation of finite-size effects at the graphene–NaCl(aq) interface. (a) Density profiles of water oxygen atoms, Cl^- ions, and Na^+ ions at the graphene–NaCl(aq) interface for a 2 M NaCl solution with different lateral dimensions. (b) Theoretical $\mathrm{Im}(\chi^{(2)}_{\mathrm{total}})$ VSFG spectra computed for the graphene–NaCl(aq) interface for the two lateral cell sizes, showing nearly indistinguishable spectral responses. (c) Top: distribution of hydrogen-bond topologies (DDAA, DDA, DAA, and DA) within the topmost interfacial water layer. Bottom: average number of hydrogen bonds per interfacial water molecule, decomposed into total, intralayer (within the first layer), and interlayer (between the first and second layers) contributions.

In the main text, we performed simulations with slit widths of ~ 20 Å. Here, we present additional results for a 1 M NaCl solution using a wider slit to assess the emergence of a bulk-like reference region. Specifically, we simulated a 1 M aqueous slit pore with a total width of 29.21 Å, as determined using the piston approach described in Section S1, and lateral dimensions of 19.760 Å × 21.390 Å. The resulting density profiles are shown in Figure S16. We find that both this larger slit and the slit widths analyzed previously exhibit a clear bulk-like region at the mid-plane, consistent with prior studies indicating that such

geometries are sufficient to recover bulk-like solution behavior.[S22,S23]

While the molar concentration quoted in the manuscript refers to the nominal bulk concentrations, these new simulations allow us to quantify local interfacial ion accumulation directly. Specifically, from the ion density profiles, we define a bulk reference concentration from the mid-plane region of the slit and quantify an interfacial enrichment factor as the ratio of the near-interface maximum to the bulk value. As shown in Figure S16, both Na^+ and Cl^- exhibit a clear enhancement near the interface, with enrichment factors of approximately ~ 3 for Na^+ and ~ 6 for Cl^-. The larger system ($W/2 = 14.605$ Å, with W being the slit width) displays a well-defined bulk plateau, confirming that this enrichment is not a finite-size artifact. This analysis provides a direct simulation-based measure of local interfacial ion accumulation relative to a bulk reference, rather than an integrated interfacial population.

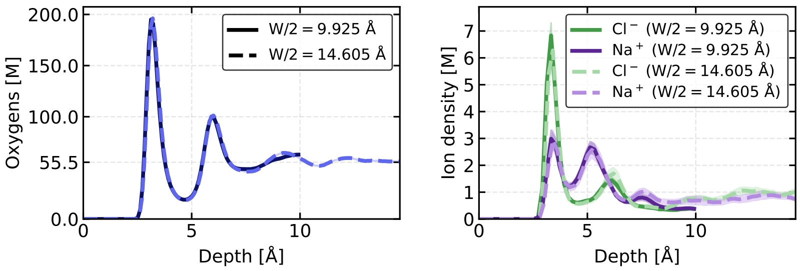

FIG. S16. Density profiles of water oxygen atoms (left) and Cl^- and Na^+ ions (right) at the graphene–NaCl(aq) interface for a 1 M NaCl solution with different slit widths. The shaded regions indicate the standard error estimated from 20 independent simulations of 200 ps each. W represents the slit width.

S6. TEMPERATURE-DEPENDENT THERMODYNAMICS OF ION ADSORPTION AT THE GRAPHENE–WATER INTERFACE

In the main text, we discuss our results with a primary focus on the graphene–NaCl(aq) interface. To further elucidate the thermodynamic origins of ion adsorption and place our conclusions on a more explicit thermodynamic footing, we carried out additional simulations at 325, 350, 375, and 400 K. From these simulations, we compute the adsorption free energy, ΔF, defined as the difference between the free energy minimum associated with the interfacial layer and a bulk-like reference region taken at the midpoint of the simulation cell. This definition provides a direct measure of the thermodynamic preference of ions to reside at the interface versus the interior of the aqueous phase. The resulting temperature-dependent free-energy profiles, together with the extracted values of ΔF for both Na^+ and Cl^-, are shown in Figure S17. At 300 K, the free-energy differences are $\Delta F = 1.65 \pm 0.04$ kcal/mol for Cl^- and 1.11 ± 0.05 kcal/mol for Na^+. These values are comparable to those obtained for H_3O^+ in a similar setup[S24] .

Figure S17 shows that the adsorption free energies of both ions vary systematically with temperature. To rationalize these trends, we decompose the free energy into enthalpic (ΔH) and entropic (ΔS) contributions. This is achieved by performing a linear fit of the temperature dependence according to $\Delta F = \Delta H - T\Delta S$, assuming that both ΔH and ΔS are approximately temperature independent over the investigated range and neglecting pressure–volume work contributions under ambient conditions. This decomposition provides direct insight into the thermodynamic driving forces governing ion stability at the interface. The resulting enthalpic and entropic contributions are shown in Figure S18.

For Cl^-, we obtain $\Delta H = -0.24 \pm 0.22$ kcal/mol and $\Delta S = 4.68 \pm 0.66$ cal/mol/K. The extracted enthalpic contribution is small in magnitude and comparable to its uncertainty, indicating that Cl^- adsorption is not driven by a strong energetic stabilization but instead reflects an entropy-dominated thermodynamic balance. As shown by some of us previously,[S1] Cl^- reduces its coordination number by approximately one when moving from the center of the slit to the interfacial region. We attribute the resulting positive entropic contribution to the release of water molecules that were previously constrained by hydrogen bonding to the anion. Concerning the enthalpic term, we speculate that the reduction in Cl^- hydration is largely compensated by the favorable Cl^-–graphene interactions.

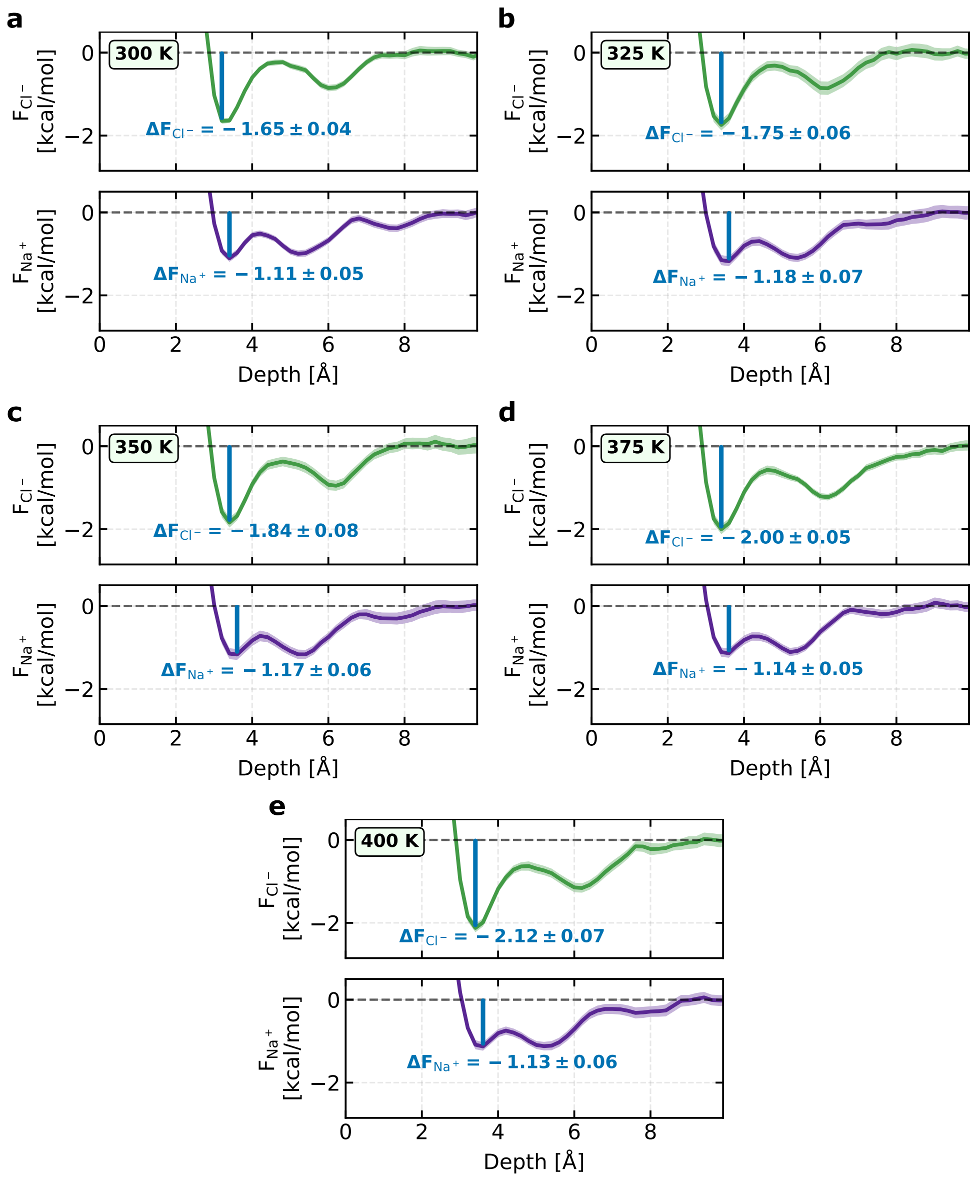

FIG. S17. Temperature dependence of ion free-energy profiles at the graphene–NaCl(aq) interface in a 0.5 M NaCl solution. Free energy as a function of depth for Cl^- (top panels, green) and Na^+ (bottom panels, purple) at temperatures of (a) 300 K, (b) 325 K, (c) 350 K, (d) 375 K, and (e) 400 K. The depth coordinate is measured perpendicular to the confining surfaces. The blue vertical markers indicate the location of the free-energy minimum relative to the bulk-like reference region, and the corresponding stabilization free energies ΔF are reported with their statistical uncertainties. The dashed horizontal lines denote the bulk reference level. The shaded regions indicate the standard error estimated from 20 independent simulations of 200 ps each.

For Na^+, we obtain $\Delta H = -1.10 \pm 0.24$ kcal/mol and $\Delta S = 0.12 \pm 0.69$ cal/mol/K. In contrast to Cl^-, the entropic contribution in this case is negligible, and interfacial adsorption is predominantly enthalpically driven. Na^+ also loses approximately one water molecule

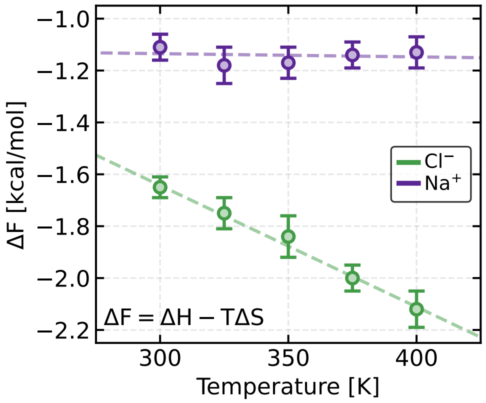

FIG. S18. Adsorption free energies of Na^+ and Cl^- at the interface as a function of temperature, together with linear fits used to extract enthalpic and entropic contributions.

when moving from the bulk to the interface. However, because cation–water interactions are weaker than anion–water interactions, the associated entropic and energetic penalties are correspondingly smaller. As a result, the graphene–Na^+ interactions dominate the interfacial stabilization.

REFERENCES

[S1] K. D. Fong, B. Sumić, N. O'Neill, C. Schran, C. P. Grey, and A. Michaelides, "The interplay of solvation and polarization effects on ion pairing in nanoconfined electrolytes," Nano Letters **24**, 5024–5030 (2024).

[S2] J. P. Perdew, K. Burke, and M. Ernzerhof, "Generalized gradient approximation made simple," Physical Review Letters **77**, 3865–3868 (1996).

[S3] S. Grimme, J. Antony, S. Ehrlich, and H. Krieg, "A consistent and accurate ab initio parametrization of density functional dispersion correction (dft-d) for the 94 elements h-pu," The Journal of Chemical Physics **132**, 154104 (2010).

[S4] M. J. Gillan, D. Alfè, and A. Michaelides, "Perspective: How good is dft for water?" The Journal of Chemical Physics **144**, 130901 (2016).

[S5] O. Marsalek and T. E. Markland, "Quantum dynamics and spectroscopy of ab initio liquid water: The interplay of nuclear and electronic quantum effects," The Journal of Physical Chemistry Letters **8**, 1545–1551 (2017).

[S6] K. D. Fong, C. P. Grey, and A. Michaelides, "On the physical origins of reduced ionic conductivity in nanoconfined electrolytes," ACS Nano **19**, 13191–13201 (2025).

[S7] A. Singraber, J. Behler, and C. Dellago, "Library-based lammps implementation of high-dimensional neural network potentials," Journal of Chemical Theory and Computation **15**, 1827–1840 (2019).

[S8] A. P. Thompson, H. M. Aktulga, R. Berger, D. S. Bolintineanu, W. M. Brown, P. S. Crozier, P. J. in 't Veld, A. Kohlmeyer, S. G. Moore, T. D. Nguyen, R. Shan, M. J. Stevens, J. Tranchida, C. Trott, and S. J. Plimpton, "Lammps - a flexible simulation tool for particle-based materials modeling at the atomic, meso, and continuum scales," Computer Physics Communications **271**, 108171 (2022).

[S9] I.-C. Yeh and M. L. Berkowitz, "Ewald summation for systems with slab geometry," The Journal of Chemical Physics **111**, 3155–3162 (1999).

[S10] L. X. Dang, "Mechanism and thermodynamics of ion selectivity in aqueous solutions of 18-crown-6 ether: A molecular dynamics study," Journal of the American Chemical Society **117**, 6954–6960 (1995).

[S11] T. Werder, J. H. Walther, R. L. Jaffe, T. Halicioglu, and P. Koumoutsakos, "On the water-carbon interaction for use in molecular dynamics simulations of graphite and carbon

nanotubes," The Journal of Physical Chemistry B **107**, 1345–1352 (2003).

[S12] N. O'Neill, B. X. Shi, K. Fong, A. Michaelides, and C. Schran, "To pair or not to pair? machine-learned explicitly-correlated electronic structure for nacl in water," The Journal of Physical Chemistry Letters **15**, 6081–6091 (2024).

[S13] D. M. Wilkins, D. E. Manolopoulos, and L. X. Dang, "Nuclear quantum effects in water exchange around lithium and fluoride ions," The Journal of Chemical Physics **142**, 064509 (2015).

[S14] D. M. Wilkins, D. E. Manolopoulos, S. Pipolo, D. Laage, and J. T. Hynes, "Nuclear quantum effects in water reorientation and hydrogen-bond dynamics," The Journal of Physical Chemistry Letters **8**, 2602–2607 (2017).

[S15] T. Ohto, K. Usui, T. Hasegawa, M. Bonn, and Y. Nagata, "Toward ab initio molecular dynamics modeling for sum-frequency generation spectra; an efficient algorithm based on surface-specific velocity-velocity correlation function," The Journal of Chemical Physics **143**, 124702 (2015).

[S16] F. Tang, T. Ohto, T. Hasegawa, W. J. Xie, L. Xu, M. Bonn, and Y. Nagata, "Definition of free o–h groups of water at the air–water interface," Journal of Chemical Theory and Computation **14**, 357–364 (2018).

[S17] T. Ohto, M. Dodia, J. Xu, S. Imoto, F. Tang, F. Zysk, T. D. Kühne, Y. Shigeta, M. Bonn, X. Wu, and Y. Nagata, "Accessing the accuracy of density functional theory through structure and dynamics of the water–air interface," The Journal of Physical Chemistry Letters **10**, 4914–4919 (2019).

[S18] K.-Y. Chiang, T. Seki, C.-C. Yu, T. Ohto, J. Hunger, M. Bonn, and Y. Nagata, "The dielectric function profile across the water interface through surface-specific vibrational spectroscopy and simulations," Proceedings of the National Academy of Sciences **119**, e2204156119 (2022).

[S19] Y. Wang, F. Tang, X. Yu, T. Ohto, Y. Nagata, and M. Bonn, "Heterodyne-detected sum-frequency generation vibrational spectroscopy reveals aqueous molecular structure at the suspended graphene/water interface," Angewandte Chemie International Edition **63**, e202319503 (2024).

[S20] C.-S. Hsieh, R. K. Campen, M. Okuno, E. H. G. Backus, Y. Nagata, and M. Bonn, "Mechanism of vibrational energy dissipation of free oh groups at the air–water interface," Proceedings of the National Academy of Sciences **110**, 18780–18785 (2013).

[S21] J. B. Asbury, T. Steinel, K. Kwak, S. A. Corcelli, C. P. Lawrence, J. L. Skinner, and M. D. Fayer, "Dynamics of water probed with vibrational echo correlation spectroscopy," The Journal of Chemical Physics **121**, 12431–12446 (2004).

[S22] S. Ruiz-Barragan, D. Muñoz-Santiburcio, and D. Marx, "Nanoconfined water within graphene slit pores adopts distinct confinement-dependent regimes," The Journal of Physical Chemistry Letters **10**, 329–334 (2019).

[S23] X. R. Advincula, C. Schran, and A. Michaelides, "When is nanoconfined water different from interfacial water?" Faraday Discussions , Accepted Manuscript (2026).

[S24] X. R. Advincula, K. D. Fong, A. Michaelides, and C. Schran, "Protons accumulate at the graphene–water interface," ACS Nano **19**, 17728–17737 (2025).
